# Genomic variations define divergence of water/wildlife-associated *Campylobacter jejuni* niche specialists from common clonal complexes

**DOI:** 10.1111/j.1462-2920.2011.02461.x

**Published:** 2011-03-21

**Authors:** Philip J Hepworth, Kevin E Ashelford, Jason Hinds, Katherine A Gould, Adam A Witney, Nicola J Williams, Howard Leatherbarrow, Nigel P French, Richard J Birtles, Chriselle Mendonca, Nick Dorrell, Brendan W Wren, Paul Wigley, Neil Hall, Craig Winstanley

**Affiliations:** 1 Institute of Infection and Global Health, St. George's, University of LondonCranmer Terrace, London SW17 0RE, UK; 2 Institute of Integrative Biology, St. George's, University of LondonCranmer Terrace, London SW17 0RE, UK; 3 National Centre for Zoonosis Research, University of LiverpoolLiverpool L69 3GA, UK; 4 Division of Cellular & Molecular Medicine, St. George's, University of LondonCranmer Terrace, London SW17 0RE, UK; 5 Massey UniversityPalmerston North, New Zealand; 6 Department of Infectious and Tropical Diseases, London School of Hygiene and Tropical MedicineKeppel Street, London WC1E 7HT, UK

## Abstract

Although the major food-borne pathogen *Campylobacter jejuni* has been isolated from diverse animal, human and environmental sources, our knowledge of genomic diversity in *C. jejuni* is based exclusively on human or human food-chain-associated isolates. Studies employing multilocus sequence typing have indicated that some clonal complexes are more commonly associated with particular sources. Using comparative genomic hybridization on a collection of 80 isolates representing diverse sources and clonal complexes, we identified a separate clade comprising a group of water/wildlife isolates of *C. jejuni* with multilocus sequence types uncharacteristic of human food-chain-associated isolates. By genome sequencing one representative of this diverse group (*C. jejuni* 1336), and a representative of the bank-vole niche specialist ST-3704 (*C. jejuni* 414), we identified deletions of genomic regions normally carried by human food-chain-associated *C. jejuni*. Several of the deleted regions included genes implicated in chicken colonization or in virulence. Novel genomic insertions contributing to the accessory genomes of strains 1336 and 414 were identified. Comparative analysis using PCR assays indicated that novel regions were common but not ubiquitous among the water/wildlife group of isolates, indicating further genomic diversity among this group, whereas all ST-3704 isolates carried the same novel accessory regions. While strain 1336 was able to colonize chicks, strain 414 was not, suggesting that regions specifically absent from the genome of strain 414 may play an important role in this common route of *Campylobacter* infection of humans. We suggest that the genomic divergence observed constitutes evidence of adaptation leading to niche specialization.

## Introduction

*Campylobacter* species are fastidious bacteria with relatively small genomes and are usually adapted to the gastrointestinal tract of various mammalian and avian hosts, primarily as commensal organisms. Yet infection due to *Campylobacter* sp., especially *C. jejuni*, is the major cause of food-borne bacterial gastroenteritis in humans worldwide (Humphrey *et al*., 2007). In addition, some patients develop post-infection complications including serious neurological disorders such as Guillain–Barré Syndrome (Yuki, 2001). It is believed that zoonotic transmission of *C. jejuni* to humans occurs primarily through the consumption and handling of livestock, with poultry being the most common source (Sheppard *et al*., 2009a). However, *C. jejuni* has been isolated from diverse animal, human and environmental sources. The reasons for virulence in humans but not livestock are unclear, but both bacterial and host factors may contribute (Zilbauer *et al*., 2008).

Factors influencing the ability of *C. jejuni* to colonize specific hosts or survive in environmental niches are poorly understood. Several studies have sought to determine the prevalence of specific clones among *C. jejuni* isolates from diverse sources by applying multilocus sequence typing (MLST) (Dingle *et al*., 2001; 2005; Colles *et al*., 2003; Manning *et al*., 2003; Sails *et al*., 2003; French *et al*., 2005; McCarthy *et al*., 2007; Karenlampi *et al*., 2007a; Taboada *et al*., 2008; Wilson *et al*., 2008; Sheppard *et al*., 2009a).While some MLST clonal complexes, such as the ST-21 complex, are widespread, others, such as the ST-61 complex, have a more restricted distribution (Colles *et al*., 2003; French *et al*., 2005). Although generally considered to be poor survivors outside of their animal hosts, some *C. jejuni* appear to be more able to survive and persist in environmental niches. In a study of *C. jejuni* in a specific area of cattle farmland in the UK (French *et al*., 2005), isolates from the ST-45 complex were much more frequently isolated from environmental water than other common clonal complexes. In addition, a number of novel sequence types not identified among human isolates have been identified from both environmental water and wildlife (birds/rabbits) sources (French *et al*., 2005; Levesque *et al*., 2008). These water/wildlife (WW) isolates represent a novel but diverse *C. jejuni* group whose distribution is consistent with transmission between wildlife and water of strains that might be adapted to survival in such niches. In addition, a second group of isolates specifically associated with bank voles (*Myodes glareolus*), characterized by a novel sequence type (ST-3704), and clonal according to pulsed-field gel electrophoresis, has been identified (Williams *et al*., 2010). The diverse WW group and clonal ST-3704 group may represent niche specialists with restricted range, in contrast to ‘generalists’ such as the ST-45 complex, which is adapted to diverse environmental, animal and human niches

The relatively small size of the *C. jejuni* genome is indicative of genomic reduction reflecting a lifestyle closer to parasitic rather than free-living bacteria (Moran, 2002). Genome sequences have been published for the *C. jejuni* strains NCTC11168 (Parkhill *et al*., 2000), RM1221 (Fouts *et al*., 2005), 81-176 (Hofreuter *et al*., 2006), 81116 (Pearson *et al*., 2007) and CG8486 (Poly *et al*., 2007). Genome sequences have also been published for strains of *Campylobacter lari*, *C. coli* and *C. upsaliensis* (Fouts *et al*., 2005), and further genome sequence projects for several other *Campylobacter* strains are ongoing. There has been considerable interest in characterizing genetic variation between isolates of *C. jejuni* with a view to identifying those genes relevant to severity of disease, host colonization or niche specialization. Inter-strain variations in loci such as those encoding lipooligosaccharide (LOS) (Karlyshev *et al*., 2005), capsule (Karlyshev *et al*., 2005), flagellin glycosylation or restriction–modification (RM) systems (Miller *et al*., 2005) have already been characterized. Comparative genome analyses using microarrays, based largely upon the genome of strain NCTC11168 (Dorrell *et al*., 2001; Leonard *et al*., 2003; Pearson *et al*., 2003; On *et al*., 2006), indicate high levels of genome diversity but low levels of genome plasticity in *C. jejuni* (Dorrell *et al*., 2005). These studies have identified discrete regions of diversity within the *C. jejuni* pan-genome, called plasticity regions PR1–PR7 (Pearson *et al*., 2003) or hypervariable regions 1–16 (Taboada *et al*., 2004; Hofreuter *et al*., 2006; Parker *et al*., 2006). Thus, although *C. jejuni* has a relatively small genome, it carries significant levels of variation, potentially indicative of evolution leading to niche specialization. A limitation of MLST is that differences in accessory genomes that may contribute to specialized host adaptation are not identified. It has been suggested that comparative genome analyses can help to identify genetic markers predictive of the source of an infection (Champion *et al*., 2005). However, genes contributing to plasticity among *C. jejuni* populations have also been identified from the genomes of strains such as RM1221, 81-176, ATCC43431 and 81116 (Pearson *et al*., 2003; 2007; Poly *et al*., 2004; 2005; Fouts *et al*., 2005), emphasizing the limitations of an approach based on a single *C. jejuni* genome. These additional genomes also indicate a role for prophages and other integrated genomic elements in strain divergence (Fouts *et al*., 2005).

Significantly, our knowledge of genomic diversity in *C. jejuni* is based exclusively on human or human food-chain-associated isolates. Thus it is likely that we do not have the true picture of the diversity of the species required to provide a genetic framework to analyse the population structure of the species. This restricts our ability to fully study the epidemiology, ecology and evolution of *C. jejuni* necessary, for example, to ascertain whether evolution of the *C. jejuni* genome may be driven by the necessity to adapt to different host and environmental niches. In this study, we report the use of a pan-genome microarray to screen a collection of 80 isolates, representing common clonal complexes and diverse sources, but including representative WW isolates. We also report the use of comparative genomic hybridization (CGH) and clustering analysis to identify a distinct clade of WW isolates, characterized by the absence of genes common among other isolates of *C. jejuni*, including genes implicated in virulence and chicken colonization. Furthermore, we report the use of whole-genome shotgun sequencing of representatives of the WW and ST-3704 groups to identify novel replacement/divergent genomic regions, and the application of indicative PCR assays to study their distribution. We demonstrate for the first time that the WW and ST-3704 isolates harbour substantial diversity in *C. jejuni*, enabling us to considerably enlarge the *C. jejuni* pan-genome, and that they are divergent from *C. jejuni* commonly associated with human infections.

## Results and discussion

### Comparative genomic hybridization

Eighty strains of diverse origin, including isolates from human, cattle, chicken, sheep, wild birds, rabbits, badgers and environmental water, were selected for analysis by CGH using the pan-genome *C. jejuni* microarray (Table S1). The strain panel, representing a more diverse selection than previous studies, was chosen to search for evidence of source-associated genes among members of widely distributed clonal complexes, in particular the ST-21 complex (*n* = 28). Isolates from other common human infection-associated clonal complexes (ST-42, ST-45, ST-48, ST-257), including a clonal complex associated with cattle (ST-61), isolates of rarer sequence types and two *C. coli*, were included in the study. For most of the clonal complex groups, there were multiple sources. In addition, eight representatives of the diverse WW group and one representative of the clonal ST-3704 were analysed.

**Figure 1:**
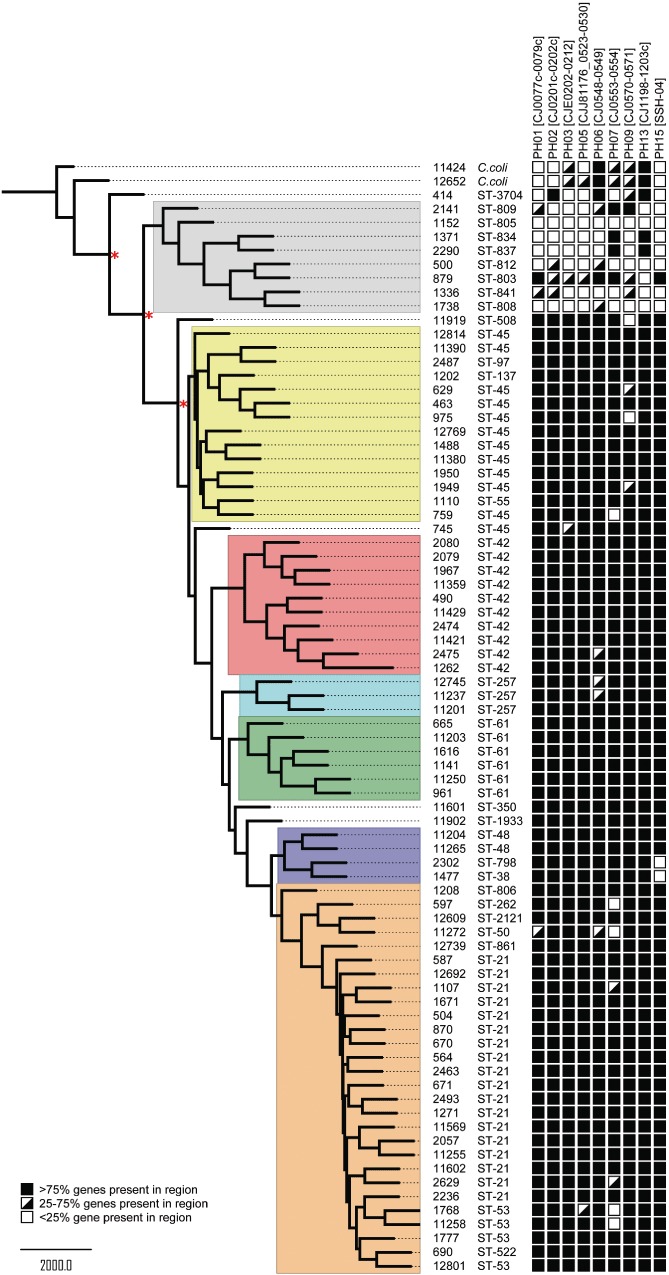
Dendrogram showing clustering of *Campylobacter* strains based on CGH. Members of the same clonal complex are shaded in the same colour, as are members of the water/wildlife group (grey). The distributions of gene clusters associated with variable regions of *Campylobacter* genomes are shown. Red asterisks indicate bootstrap values > 95% for the main clades.

A dendrogram based on phylogenomic cluster analysis of the CGH data revealed that strains generally clustered according to clonal complex, and there was no clear evidence for subdivisions according to isolate source within the clonal complexes (Fig. 1). Most strikingly, the eight WW group isolates included in the study formed a distinct clade. In addition, strain 414, representing ST-3704, was also genetically distinct from the food-chain-associated clonal complexes, clustering between the WW group and *C. coli* isolates (Fig. 1). It was clear from the CGH data that the separation of WW/ST-3704 isolates was due to a number of genes or gene clusters where deletion or divergence was apparent (Fig. 1). Regions of divergence or deletions associated with the WW/ST-3704 isolates are listed in Table S2 (PH01–PH15). The distribution of nine regions of divergence, which included the virulence-associated cytolethal distending toxin (*cdt*) genes (Dorrell *et al*., 2001; Taboada *et al*., 2004; Parker *et al*., 2006), is shown in Fig. 1. The regions chosen for inclusion in the figure are those generally conserved among clonal complexes commonly associated with isolates from human infections, but highly diverse among the WW/ST-3704 isolates.

In a previous study using CGH, *C. jejuni* isolates were separated into two distinct clades: a ‘livestock’- and a ‘non-livestock’-associated clade (Champion *et al*., 2005). We further compared the isolates in this study with those used in the previous study of Champion and colleagues (2005). The clustering obtained indicated that the WW isolates clustered most closely with isolates from the previously reported non-livestock clade, in particular the environmental beach isolates 1791, 1792 and 1793 (Fig. S1). Many of the isolates from the previously reported ‘non-livestock’ clade clustered with the WW isolates and the ST-45 complex. It is notable that the ST-45 complex is more often isolated from environmental sources than other common clonal complexes. However, other ‘non-livestock’ clade isolates clustered with members of the ST-42, ST-61, ST-257 and ST-48 complexes.

Comparative phylogenomics by microarray analysis is potentially a strong tool for the study of population structures leading to insights into pathogen evolution and divergence in relation to virulence and host preferences (Dorrell *et al*., 2005). MLST has been used to demonstrate the relative importance of different sources of *Campylobacter* in the context of human infections (Sheppard *et al*., 2009a,b; Strachan *et al*., 2009). However, it has been suggested that analysis of full allelic sequences, rather than simple MLST, can better predict host association for isolates from cattle or sheep (McCarthy *et al*., 2007). Others have suggested that although clustering of *C. jejuni* according to MLST does in general agree with clustering by CGH, genomic mosaicism between closely related strains may lead to significant phenotypic variations undetectable by MLST (Taboada *et al*., 2008). Overall, our observations are consistent with the notion that while MLST can give valuable population data, detailed characterization of the factors influencing bacterial host preferences and pathogenicity can only be achieved by studying genomic variations on a larger scale. In order to address this further for the novel clade of isolates identified in this study, we selected two representative strains for further study (1336 and 414).

### Colonization of chickens

**Figure 2:**
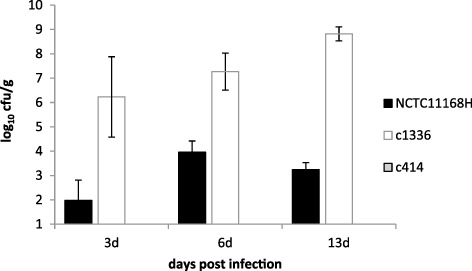
Caecal colonization of 3-week-old SPF Light Sussex chickens following experimental infection with *C. jejuni* strains NCTC11168H, 1336 and 414. Data shown are the mean (±SEM) based on five birds per group at each time point (414 counts were all < 1).

Both the *C. jejuni* control strain, NCTC11168H, and strain 1336 colonized the caeca of experimentally infected chickens, whereas strain 414 was unable to colonize (Fig. 2). Isolate 1336 colonized to significantly higher levels than NCTC11168H at both 6 days (*P* = 0.006) and 13 days (*P* < 0.001) post infection. None of the isolates was found to be invasive. Both NCTC11168H and 1336 caused mild inflammation to the ileum and caeca. This was slightly more evident with 1336, and was accompanied by mild diarrhoea, probably reflecting the higher level of colonization found in these birds.

### Genome sequencing of *C. jejuni* strains 1336 and 414

**Figure 3:**
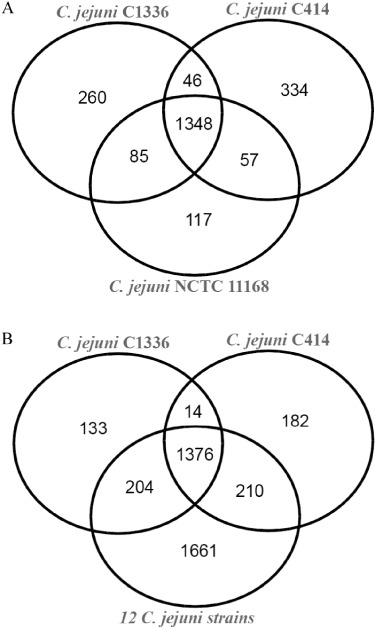
Venn diagrams showing orthologues shared by strains by 1336 and 414 with (A) *C. jejuni* NCTC11168 only and (B) 12 *C. jejuni* strains.

In order to further investigate the divergence between WW/ST-3704 isolates and common human-associated isolates, we shotgun sequenced the genomes of strains 1336 (ST-841; a representative of the WW group, chosen because CGH indicated it as one of the most divergent from NCTC11168) and 414 (ST-3704). A basic summary of the sequencing data is shown in Table S3. When compared with the genome of *C. jejuni* NCTC11168 and each other, the genomes of strains 1336 and 414 harboured 260 and 334 unique ORFs respectively (Fig. 3A). These numbers were further reduced to 133 and 182, respectively, when a comparison was extended to include 12 genome sequenced *C. jejuni* strains (Fig. 3B).

### Deletions and divergence in the genomes of the *C. jejuni* ST-841 and ST-3704 strains

Using the ACT tool, we compared the pseudogenomes of strains 1336 and 414 with the genome of strain NCTC11168. A summary of all of the regions of major divergence is provided in Table S4 and comparison files to view the genome alignments will be made available on request. For each of the previously reported seven plasticity regions (Pearson *et al*., 2003), or 16 regions of divergence (Taboada *et al*., 2004; Parker *et al*., 2006) defined with respect to the genome of strain NCTC11168, there was evidence of deletions or divergence in the genomes of both strains 1336 and 414 (Table S4). These include regions associated with LOS, capsule, flagellin glycosylation, motility, iron transport, other transport systems and RM.

A region within the flagellin glycosylation locus (Cj1321–Cj1325) has been identified as characteristic of a ‘livestock’ clade (Champion *et al*., 2005). Based on the presence or absence/divergence of these ORFs according to CGH analysis, we found evidence that this cluster was divergent in a clonal complex-dependent manner (Table S1). The genes were present in the ST-21 and ST-48 complexes, regardless of source, but the locus was highly divergent in the ST-42, ST-61 and ST-257 complexes. The absence/divergence was variable in the ST-45 complex and in the WW group (Table S1). The genomes of strains 1336 and 414 also differed with respect to the Cj1321–Cj1325 locus, which was entirely deleted from the genome of strain 1336, but only partially deleted (Cj1321–Cj1323) from the genome of strain 414. This partial deletion was confirmed by PCR assay in seven different ST-3704 isolates. The same PCR assay indicated that the deletion in three of 10 WW group isolates tested may also only be partial. In addition, we used a PCR assay to confirm the presence of a divergent strain 414 gene equivalent of Cj1324 in each of seven ST-3704 isolates (data not shown). Others have not observed a clear association between genes from this locus (the Cj1321 and Cj1324 genes) and livestock-associated isolates (Karenlampi *et al*., 2007b). In a recent study it has been shown that the Cj1321–Cj1325 genes are responsible for a legionaminic acid modification of the glycosylated flagellin, and that a Cj1324 mutant is less able to colonize and persist in chickens (Howard *et al*., 2009). However, the fact that some chicken isolates in our study lack Cj1321–Cj1325 supports the notion that multiple factors are involved in colonization.

There were a number of deletions from the genomes of strains 1336 and 414 in regions associated with host–bacterial interactions, including the ability to colonize chickens (Table 1). Notably the *cdtABC* cluster was completely deleted from the genome of strain 414, while the *cdtA* gene only was deleted from the genome of strain 1336, which also lacks the adhesin-encoding *peb3* gene (Min *et al*., 2009). The Cj0055–Cj0058 region includes Cj0057, reported as downregulated during chick colonization (Woodall *et al*., 2005). Although the four ORFs are deleted from the genome of strain 414, strain 1336 retains equivalent ORFs to Cj0057 and Cj0058. Our CGH data indicated a variable distribution for Cj0055, Cj0056 and Cj0058, but notably not Cj0057, among ST-45 complex strains. Cj0057 and Cj0058, a putative peptidase, are also included in a list of divergent genes associated with the ST-45 complex in the recent study of Taboada and colleagues (2008). Each of the iron transport-related ORFs Cj0177–Cj0181, also reported as divergent in ST-45 isolates (Taboada *et al*., 2008), were absent/divergent according to our CGH data for multiple strains from ST-45, ST-42 and the WW group, including two ST-45 chicken isolates, despite the reported reduced colonization for a Cj0178 mutant (Palyada *et al*., 2004). The regions Cj0423–Cj0425 and Cj0617–Cj0618 were also not solely absent/divergent in WW/ST-3704 isolates, whereas for Cj0437–Cj0439, Cj0453, Cj0830, Cj0903, Cj1183 divergence was only detected in ST-3704. According to the CGH analysis, Cj0628, encoding the putative autotransporter CapA, was absent/divergent in 25 strains, including nine of 10 ST-42 isolates, six of seven ST-61 isolates and six of 14 ST-45 isolates, including both chicken isolates. The latter is notable because of the reported attenuation of chick colonization for a *capA* mutant (Ashgar *et al*., 2007). ORFs from the cluster Cj1721–Cj1727 (but not Cj1725) were absent/divergent by CGH for multiple strains.

**Table 1:** Regions of divergence in strain 1336 (WW) and strain 414 (ST-3704) genomes implicated in host–bacterial interactions.

NCTC11168 location	Comments	Reference
Cj0055–Cj0058^a^	Deleted from 414, divergent in 1336; Cj0057 downregulated during chick colonization	Woodall *et al*. (2005)
Cj0077–Cj0079	*cdtABC* partly deleted from 1336, totally deleted from 414 (cytolethal distending toxin)	Zheng *et al*. (2008)
Cj0177–Cj0181^a^	Deleted from both; iron transport; mutant has reduced colonization in chick model	Palyada *et al*. (2004)
Cj0288–Cj0300^a^	Large divergent region in both 1336 and 414; includes *peb3* and *panBC*, downregulated during chick colonization; 1336 contains putative alternative *cdtABC*-like cluster	Woodall *et al*. (2005)
Cj0414–Cj0415	Divergent in 414; putative oxidoreductase subunits in NCTC11168; Cj0414/0415 reported as downregulated during chick colonization; Cj0414 required for chicken colonization	Woodall *et al*. (2005); Pajaniappan *et al*. (2008)
Cj0423–Cj0425	Divergent in both; membrane proteins; Cj0425 downregulated during chick colonization	Woodall *et al*. (2005)
Cj0437–Cj0439	Divergent in 414; upregulated during chick colonization	Woodall *et al*. (2005)
Cj0453	Divergent in 414; downregulated during chick colonization	Woodall *et al*. (2005)
Cj0617–Cj0618^a^	Deleted from 1336; Cj0618 mutant attenuated for chick colonization	Hendrixson and DiRita (2004)
Cj0628	Deleted/divergent in both; CapA autotransporter downregulated during chick colonization; *capA* mutant attenuated for chick colonization	Woodall *et al*. (2005); Ashgar *et al*. (2007)
Cj0755	Deleted from both; ferric enterobactin receptor (CfrA); mutant unable to colonize GI tract in chick model	Palyada *et al*. (2004)
Cj0830	Deleted from 414; membrane protein, downregulated during chick colonization	Woodall *et al*. (2005)
Cj0864–Cj0866	Divergent in both; upregulated during chick colonization	Woodall *et al*. (2005)
Cj0873–Cj0878	Divergent in 414; Cj0874 & Cj0876 downregulated during chick colonization	Woodall *et al*. (2005)
Cj0903	Deleted from 414; transport protein, mutation attenuated for chick colonization	Hendrixson and DiRita (2004)
Cj0987–Cj0990	Divergent in both; membrane proteins/transport; Cj0987 downregulated during chick colonization	Woodall *et al*. (2005)
Cj1135–Cj1149	Divergent in both; LOS locus	Perera *et al*. (2007)
Cj1183	Deleted from 414; downregulated during chick colonization	Woodall *et al*. (2005)
Cj1316–Cj1341	Divergent in both; glycosylation locus; includes insertion in 1336; Cj1321–Cj1325 identified as characteristic to livestock isolates	Champion *et al*. (2005)
Cj1415–Cj1442	Divergent in both; capsule locus	Bachtiar *et al*. (2007)
Cj1667–Cj1668^a^	Divergent in both; Cj1668 downregulated during chick colonization	Woodall *et al*. (2005)
Cj1721–Cj1727	Deleted/divergent in both; Cj1725 (deleted from both) downregulated during chick colonization	Woodall *et al*. (2005)

a Divergence reported previously, mainly associated with the ST-45 complex (Taboada *et al*., 2008).

In order to analyse the distribution of deleted regions, PCR assays were designed targeting ORFs flanking six of the deleted regions, including the *cdt* genes and the Cj0177–Cj0181 region. These assays were used to screen a panel of strains including 10 WW group isolates, seven ST-3704 isolates, and representatives of common human-associated clonal complexes. Each of the deletions was confirmed in between six and nine WW group isolates, with the minority isolates differing in each case, suggesting considerable variation within this group. The ST-3704 isolates all shared the same deletions as each other, albeit not always the same size of deletion as the WW group (Table 2).

**Table 2:** Summary table of PCR distributions.

								Number of PCR positives for novel regions^a^																			
	Confirmed deletions							Strain 1336																	414		
	*n*	552	967	818	1167	177	*cdt*^b^	1a	1b	1c	1d	2	3	4a	4b	5a	5b	5c	6	7a	7b	8a	8b	*cdt*^c^	1	2	3
WW	10	8	8	8	8	6	9	6	8	7	7	5	2	2	1	3	5	2	2	1	1	3	9	9	2	0	0
ST-3704	7	7	7	7	7	7	7	0	0	0	0	0	0	0	0	0	0	0	0	0	0	0	0	0	7	7	7
ST-21	2	0	0	0	0	0	0	0	0	0	0	0	0	0	0	0	0	0	0	0	0	0	0	0	0	0	0
ST-45	3	1	0	3	0	3	0	0	0	0	0	0	0	0	0	0	0	0	0	1	1	0	0	1	1	0	0
ST-42	3	1	0	0	0	3	0	0	0	0	0	0	0	0	0	0	0	0	0	0	0	0	0	0	1	1	0
ST-61	1	0	0	1	0	0	0	0	0	0	0	0	0	0	0	0	0	0	0	0	0	0	0	0	0	0	0
ST-257	1	0	0	1	0	0	0	0	1	1	0	0	0	0	0	0	0	0	0	0	0	0	0	0	0	0	0

a. a, b, c, d indicate multiple ORFs within the same region. Information about the specific ORFs targeted is shown in Table S6.

b. *Campylobacter jejuni cdt* gene cluster.

c. Divergent *C. lari*-like putative *cdtA.*

For strain 1336 region 2, only results for C1336_00025_106 are shown. Using the assay for C1336_00025_57, only strain 1336 itself was PCR-positive.

### Novel regions of the genomes of the *C. jejuni* WW and ST-3704 strains

In addition to deletions with respect to the genome of strain NCTC11168, there were a number of often large insertions in the genomes of strains 1336 and 414, although few were shared by the two strains. A summary of some major islands/regions of divergence is presented in Table S5, with all regions listed in Table S4.

Based on the CGH analysis, we surveyed the distribution of plasmid-associated (pVir and pTet) and integrated element-associated ORFs included on the microarray (CMLP1, CJIE2-4) (Table S1). As indicated by the CGH data (Table S1), the genomes of both strains 1336 and 414 contain integrated elements related to those described in strain RM1221 (Fouts *et al*., 2005). The genome of the WW group strain 1336 carries integrated elements related to CJIE2 and CJIE3. As in strain RM1221, the CJIE2-like element is adjacent to a tRNA_Arg_ locus between ORFs matching the strain NTCC11168 ORFs Cj0493 and Cj0494. However, the strain 1336 CJIE3-like element is not located in the same position as in RM1221 (between Cj1011 and Cj1012, adjacent to a tRNA_Arg_) relative to the genome of strain NCTC11168. In strain 1336 the CJIE3-like element is integrated between Cj0936 and Cj0937, adjacent to a tRNA_Leu_ locus. The genome of the ST-3704 strain 414 contains integrated elements related to CMLP1 and CJIE4. The Mu-like prophage CMLP1 is located between ORFs matching the NCTC11168 ORFs Cj0653 and Cj0659, whereas in strain RM1221 the element is in a different location (between Cj0223 and Cj0224 of strain NCTC11168). The CJIE4-like element of strain 414 is in the same location relative to the genome of strain NCTC11168 as in strain RM1221 (between Cj1282 and Cj1283 in 414, adjacent to a tRNA_Met_ locus). The integrated CMLP1-like element in 414 also contains a cluster of putative type VI secretion genes encoding predicted proteins sharing high levels of identity with plasmid pCC178-encoded *C. coli* RM2228 predicted proteins, including Hcp1 and VgrG family proteins (secreted effectors).

In a previous survey of 67 *C. jejuni* from various sources, but of unknown MLST type, 55% of isolates were positive for at least one RM1221-like element, while 27% were positive for two or more (Parker *et al*., 2006). Diversity in elements similar to those present in RM1221 exhibit modular patterns. Our observations are consistent with previous work showing that CMLP1- and CJIE3-like elements have variable genomic insertion points (Parker *et al*., 2006).

Other major regions of divergence, identified using both CGH and genome sequencing, included regions related to LOS, capsule and flagellin glycosylation. Variations in those genomic regions contributing to the *C. jejuni* glycome has been noted previously (Karlyshev *et al*., 2005). Novel regions containing ORFs sharing similarity with RM or iron regulation and transport were also found. Diversity in RM loci (Miller *et al*., 2005) and regions associated with iron regulation (Palyada *et al*., 2004) have also been reported previously.

Notably, within the region of the strain 1336 genome corresponding to plasticity region 1 (PR1) (Pearson *et al*., 2003), there is a cluster of genes encoding predicted proteins sharing 62–80% identity with *C. lari* putative CdtA, CdtB and CdtC (C1336_000060027–9). This region may therefore encode an alternative cytolethal distending toxin to compensate for the loss of the *C. jejuni*-like *cdtA* gene. PCR assays indicated that the alternative *cdtA* gene was widely distributed among the WW group (Table 2).

Indicative PCR assays based on ORFs from each of the regions shown in Table S5 were used to study the distribution of novel regions of the genomes of strains 1336 and 414 among a panel of isolates including multiple WW and ST-3704 isolates (Table 2). We found no evidence for genetic diversity among the seven ST-3704 isolates from different bank voles. In contrast, variation among the 10 WW group isolates was evident both in the distribution of, and within, variable regions. For example, each of the four PCR assays for 1336 region 1 yielded a different distribution among WW isolates, although there was a core set of five isolates testing PCR-positive for each assay. All WW isolates were PCR-positive for at least one of the 1336 region assays, but no other isolate (except strain 1336) was PCR-positive for all. The most similar were three of the WW isolates (500, 864 and 1371), which were PCR-positive for at least one of the assays for each of six 1336 regions. Among the other isolates tested, PCR-positives were rare for either strain 1336 or strain 414 novel regions (Table 2).

### Phylogenetic analysis of strains 1336 and 414

It has been proposed, based on patterns of genetic exchange within the seven MLST scheme housekeeping genes, that *C. jejuni* and *C. coli* are converging as a consequence of human activity leading to increased opportunities for genetic exchange (Sheppard *et al*., 2008). Using the concatenated predicted protein sequences of 12 conserved genes, we aligned isolates 1336 and 414 with other available *Campylobacter* and related species and strains (Fig. S2). Using comparisons based on the 12 genes, strain 1336 falls within the main *C. jejuni* cluster, separate from *C. coli* and other *Campylobacter* spp. The position of strain 414, between *C. jejuni* and *C. coli*, is less clear, suggesting that this strain may represent a subspecies of *C. jejuni* (Fig. S2). We also constructed a dendrogram based on orthologues (Fig. S3). This demonstrates less ability to distinguish *C. jejuni* from *C. coli.* However, the relative strain positions on Fig. S3, which may be interpreted as indicating convergence, are heavily influenced by the distribution of orthologous horizontally acquired genes, such as those carried by prophages. For example, strain 414 shares more genes with *C. coli* RM2228 than it does with *C. jejuni* NCTC11168, but it shares a greater number with *C. jejuni* RM1221, largely because of large integrated genetic elements. However, strain 414 is more divergent from most *C. jejuni* than strain 1336.

### Conclusions

The WW group represent a group of *C. jejuni* with novel sequence types, none of which has been identified among the 507 chicken isolates in the MLST database (http://pubmlst.org). Although there has been a recent addition of one human stool isolate of ST-841 associated with a sporadic infection in England (Dingle *et al*., 2008), this group is otherwise not represented among human isolates either. The single exception does suggest that ST-841 isolates, such as strain 1336, are capable of causing infections in humans. In contrast to strain 414, strain 1336 was an effective colonizer of 3-week-old chickens (Fig. 1), despite the fact that its genome lacks several genes associated with chicken colonization. This suggests that regions associated with chick colonization by *C. jejuni*, but missing from the genome of strain 1336, are not essential for the colonization phenotype. This phenotype is likely to be multifactorial and other loci may compensate for the loss. Alternatively, the function of deleted genes may have been replaced by alternative genes lying within novel regions of the 1336 genome. Hence, a gene that is essential for colonization by one strain of *C. jejuni* may not be essential for a different strain of *C. jejuni.*

It is possible that effective colonization by 1336-like strains would not occur at the levels of exposure experienced naturally by chickens. Alternatively, the lack of WW MLST types among chicken isolates could reflect very low levels of exposure to these *C. jejuni* types, either because chickens do not have access to the environmental niches occupied by the WW group, or because the WW group are present in relatively small numbers in the environment. The vertically integrated nature of the poultry industry and strict biosecurity may limit transmission between poultry and other sources, such as wild birds. Also, the nature of sampling of isolates may mean that those occurring at a lower frequency are simply not detected (Dopfer *et al*., 2008). However, those genes associated with chick colonization specifically lacking from the genome of strain 414 warrant further investigations. Interestingly, strain 414, representing a bank-vole specialist, can invade T84 human intestinal cell line and elicit an IL-8 response (H. Murphy and T. Humphrey, pers. comm.), suggesting that it may have the potential to be pathogenic against humans, but that the reservoirs of *Campylobacter* to which bank voles and humans are exposed do not overlap. Despite all the research on *C. jejuni* for the last 35 years, we still do not know if there are strains exhibiting variable or no pathogenicity to humans.

Although this study has highlighted a number of genes associated with particular hosts/niches, suggesting evidence at the genomic level of evolution leading to niche specialization, the precise mechanisms remain to be elucidated. However, we suggest that closer analysis of such isolates could provide crucial clues towards a better understanding of the factors influencing the niche adaptation and pathogenicity of *Campylobacter* spp.

## Experimental procedures

### Strain panel, growth conditions and extraction of DNA

The strains used in this study were obtained as part of a larger survey of *Campylobacter* isolates from environmental, farm animal and human sources (Table S1) and mainly comprise a subset of the isolates used in a previous study (Hepworth *et al*., 2007). The WW group, highlighted previously as the ‘water/wildlife group’ (French *et al*., 2005), were identified following the characterization of *C. jejuni* populations in a well-defined area of farmland (French *et al*., 2005; Kwan *et al*., 2008). For PCR screening, the additional WW group isolates 637 (water; ST-835) and 864 (rabbit; ST-802) were included. Strain 414 represents a novel group of bank vole isolates characterized by ST-3704 and common PFGE profiles (Williams *et al*., 2010). For microarray analysis, the reference strain NCTC11168 was used (Parkhill *et al*., 2000). Strains were maintained on blood agar incubated at 37°C in a microaerophilic atmosphere (Campygen- Oxoid). DNA was extracted from cell suspensions of bacterial cultures grown on solid media, using the Wizard Genomic DNA Purification Kit (Promega).

### Microarray analysis

The BμG@S CJv3.0.0 microarray incorporates spotted 60mer oligonucleotide reporters to represent each predicted coding sequence (CDS) in the genomes of *C. jejuni* strains NCTC11168, RM1221 and 81-176 plus additional strain-variable sequences from plasmids (Bacon *et al*., 2000; 2002), capsule, LOS and subtractive hybridizations (Hepworth *et al*., 2007). Genomic DNA extracted from the *C. jejuni* strains used in this study were labelled and hybridized to the microarrays, according to standard protocols, and subsequently scanned to capture the raw fluorescence intensity data for each reporter. Further details of the microarray methods used are provided in *Supporting information*. Fully annotated microarray data have been deposited in BμG@Sbase (Accession No.: E-BUGS-95; http://bugs.sgul.ac.uk/E-BUGS-95) and also ArrayExpress (Accession No.: E-BUGS-95).

### Clustering analysis of CGH data

For each strain analysed in this study, the presence or absence of each CDS in comparison with a *C. jejuni* NCTC11168 control was determined. The presence/absence call for each CDS was used to construct a binary matrix, from which a consensus phylogenetic tree was derived by maximum parsimony using PHYLIP (Felsenstein, 1993). An additional meta-analysis of strains in this study combined with a collection of strains previously analysed (Champion *et al*., 2005) was performed to further investigate association with source. The two data sets were generated with different microarray designs and pan-genome coverage, therefore the call of gene presence/absence was compiled for only those genes common to both data sets.

### 454 genome sequencing

*Campylobacter jejuni* strains 1336 and 414 were both sequenced using the Roche 454 Genome Sequencer FLX (GS-FLX) following the manufacturer's instructions (Roche 454 Life Science, Branford, CT, USA). In brief, each sample was made into both a paired-end and a fragment library using the standard FLX chemistry for 454. Fragment libraries were prepared by fragmentation, attachment of adapter sequences, refinement of the ends and selection of adapted molecules. Paired-end libraries were produced by hydroshear shearing, circularization, addition of adapters and selection as for the fragment library. Both libraries were amplified by emPCR and fragment-containing beads recovered and enriched. Sequencing primers were added and each library was deposited onto a quarter of a PicoTiterPlate plate and sequenced. Further details of genome assembly and annotation are provided in *Supporting information*. The Whole Genome Shotgun projects for strains 1336 and 414 have been deposited at DDBJ/EMBL/GenBank under the accessions ADGL00000000 and ADGM00000000 respectively. The versions described in this article are the first versions, ADGL01000000 and ADGM01000000.

### Phylogenetic analysis and Venn diagrams

The methods used to construct dendrograms based on concatenated predicted protein sequences of 12 conserved genes and on shared orthologues, and the construction of Venn diagrams to display overlap between genomes, are described in *Supporting information*.

### PCR-based screening

PCR amplification was carried out using purified genomic DNA as template. Details of the oligonucleotide primers used for PCR assays are given in Table S6.

### Experimental infection of chickens

Experimental infection of chickens was performed based on the methods of Jones and colleagues (2004). Forty-five 1-day-old specified pathogen-free Light Sussex chickens (Institute for Animal Health, Compton, UK) were divided into groups of 15 that were raised on the floor in individual pens and given *ad libitum* access to water and a vegetable protein-based diet (SDS, Witham, Essex, UK). Birds were maintained at a temperature of 30°C until 3 weeks of age, then at 20°C. At 22 days of age birds were inoculated orally with 10^8^ cfu of *C. jejuni* 414, *C. jejuni* 1336 and *C. jejuni* NCTC11168H the hypermotile variant previously described as colonizing chickens (Jones *et al*., 2004). Inocula were grown for 48 h in Mueller–Hinton broth at 37°C under microaerophillic conditions. At 3, 6 and 13 days post infection, five birds from each groups were killed by cervical dislocation and post-mortem analysis was performed. Samples of liver and caecal contents were removed aseptically for bacteriology and any gross pathological changes noted.

### Bacteriological analysis

Samples were placed in phosphate-buffered saline and homogenized for 2 min in a Biomaster 80 microStomacher (Seward, Worthing, UK). The homogenates were decimally serially diluted in PBS, then plated for enumeration onto CCDA agar (Oxoid, Basingstoke, UK) then incubated for 48 h under previously described conditions. Statistical comparison between groups was made through analysis of variance (anova).

## Notes

Fig S1 Dendrogram of *Campylobacter*isolates clustered according to CGH meta-analysis data comprisedfrom this study and a previous study (Champion *et al*.,2005). Isolates identified in the previous study as being part ofthe livestock or non-livestock clusters are highlighted in red andblue respectively.

Fig S2 Dendrogram of *Campylobacter* and related strains based on concatenated nucleotide sequences.

Fig S3 Dendrogram of *Campylobacter* and related strains based on predicted protein orthologues.

Table S1 Summary of microarray comparative genome hybridizations (CGH) to indicate the presence or absence/divergence of selected genomic loci and distribution among the strains.

Table S2 Regions of divergence associated with the WW/ST-3704 isolates according to CGH.

Table S3 Summary of genome sequence data.

Table S4 Novel features of the *C.jejuni* 1336 and 414 genomes in comparison with NCTC11168. The data presented are based on comparisons of genome sequence data.

Table S5 Selected novel genomic regions of strains 1336 and 414 targeted for PCR assays.

Table S6 Details of oligonucleotide primers for PCR assays.
