# Supplementary material for: Genomic variations define divergence of water/wildlife-associated Campylobacter jejuni niche specialists from common clonal complexes
Source: Environ Microbiol. 2011 Mar 21;13(6):1549–60. doi: 10.1111/j.1462-2920.2011.02461.x (PMC3569610; doi:10.1111/j.1462-2920.2011.02461.x)
Supplement: Fig S1 — Dendrogram of Campylobacterisolates clustered according to CGH meta-analysis data comprisedfrom this study and a previous study (Champion et al.,2005). Isolates identified in the previous study as being part ofthe livestock or non-livestock clusters are highlighted in red andblue respectively. [file emi0013-1549-sd1.doc]

**Supplementary Methods**

**Microarray analysis**

The collection of strains in this study were analysed by microarray comparative genome hybridisation (CGH) to survey gene presence or absence/divergence across the genome, enabling the inference of phylogeny and to investigate the possible association between gene content and source of the isolate. A *C. jejuni* pan-genome microarray, incorporating all available genome sequences at the time of design, was utilised to provide a comprehensive surveillance of gene distribution. The BµG@S CJv3.0.0 microarray was designed to provide coverage of all the annotated CDS present in the genomes of *C. jejuni* NCTC11168, RM1221 and 81-176, ideally using a single 60mer oligonucleotide reporter to represent a particular gene and its orthologues in other strains. A number of additional genes, not present within the genomes included in the design, were also included on the microarray to extend gene representation. These additional genes included plasmids, genes from the capsule or LOS loci of other strains and genes identified through subtractive hybridisation experiments. The array design is available in BµG@Sbase (accession number: A-BUGS-37; http://bugs.sgul.ac.uk/A-BUGS-37) and also ArrayExpress (accession number: A-BUGS-37).

CGH analysis by microarray was performed according to standard BµG@S protocols used in previous studies (Champion et al., 2005). Briefly, genomic DNA extracted from each strain was fluorescently labelled with Cy5 using random primers and Klenow DNA polymerase, purified and co-hybridised with a Cy3 labelled sample of *C. jejuni* 11168. Following overnight hybridisation, the microarrays were washed and then scanned in an Affymetrix 428 scanner to capture the fluorescent intensity of each oligonucleotide reporter in both channels. Feature extraction from microarray images was performed using BlueFuse for Microarrays v3.5 (BlueGnome, Cambridge, UK) and also ImaGene v5.5 (BioDiscovery, USA) software to generate raw data for further analysis. Raw data was either processed using BlueFuse, to exclude low confidence data and apply global Lowess normalisation, or using GeneSpring v7.3 (Agilent Technologies, USA) for normalisation, data visualisation and further analysis.

**Clustering of CGH data**

Microarray data was normalised using LIMMA median normalisation. The normalised microarray data were processed to determine gene presence or absence by either the BlueFuse CGH calling algorithm, using thresholds based on greater than two standard deviations of the genome and 2-fold cutoffs, or GACK software (Kim et al., 2002) using binary output with expected probability of presence thresholds set at 0.5. Consensus phylogenetic trees were derived by maximum parsimony using the PHYLIP programs seqboot, pars and consense with 1000 bootstrap replicates and 100 random re-orderings of strains. For the meta-analysis with the Champion *et al.* dataset then ImaGene raw data was used to ensure there was no experimental variation introduced due to feature extraction software. The two datasets were generated with different microarray designs and pan-genome coverage, therefore the call of gene presence/absence was compiled for only those genes common to both datasets. Fully annotated microarray data has been deposited in BµG@Sbase (accession number: E-BUGS-95; http://bugs.sgul.ac.uk/E-BUGS-95) and also ArrayExpress (accession number: E-BUGS-95).

**Genome assembly and annotation**

For each strain, reads from the fragment and paired-end libraries were de-novo assembled into contigs which in turn were incorporated into scaffolds using the Roche 454 Newbler assembler (version 2.0.01.12) with default settings. The resulting scaffolds were ordered and orientated with respect to the genome of *C. jejuni* NCTC 11168 (AL1111678.1) using an in-house Perl script and the alignment program NUCmer (part of the MUMmer 3.20 software package; <http://mummer.sourceforge.net/>). A single scaffold was then generated from this ordering with 100 base stretches of Ns separating each scaffold.

For each scaffold-contig in turn, putative ORFs were called using Glimmer version 3.02 (<http://www.cbcb.umd.edu/software/glimmer/>). A further in-house Perl script was then run to identify and correct those ORFs likely to have been split due to sequencing errors when handling homopolymer repeats. This involved BLASTP alignment of ORF protein translations against a database of translations generated from previously annotated *C. jejuni* genomes, the identifications of likely INDELS within homopolymer regions, the modification of coding sequence feature positions to correct error, and the merging of relevant ORFs. Where such modification occurred, this was recorded as metadata (in the form of the eventual GenBank feature note field). Such ORFs were also marked with the exception flag set to 'low-quality sequence region' for the final GenBank submission to signify poor quality sequencing.

Putative function was then assigned to each gene by BLASTN (NCBI Blast 2.2.17) comparison with a database of sequences generated from previously annotated *C. jejuni* genomes (*C. jejuni* subsp. *jejuni* NCTC 11168, AL11168; *C. jejuni* RM1221; CP000025; *C. jejuni* subsp*. jejuni* 81-176, CP000538; *C. jejuni* subsp.

*doylei* 269.97, CP000768; *C. jejuni* subsp. *jejuni* 81116, CP000814). Putative tRNA genes were detected using tRNAscan-SE 1.23 (<ftp://selab.janelia.org/pub/software/tRNAscan-SE/>).

Resulting annotated contigs and accompanying scaffold information were submitted as a WGS project to NCBI repository under accessions ADGL00000000 (*C. jejuni* 1336) and ADGM00000000 (*C. jejuni* 414). Additional “pseudogenomes”, consisting of a concatenatation of contigs guided by the scaffold with 100 Ns separating adjoining contigs, were prepared for comparison purposes using the programs Artemis and ACT (Carver et al., 2005; Rutherford et al., 2000). The development of Artemis and ACT was funded by the [Wellcome Trust's](http://www.wellcome.ac.uk/) Beowulf Genomics initiative, through its support of the [Pathogen Sequencing Unit](http://www.sanger.ac.uk/Teams/Pathogen/), Sanger Centre, UK.

**Phylogeny based on twelve conserved genes**

Based on the tree originally published by Fouts *et al*., (Fouts et al., 2005) a maximum likelihood phylogeny based on twelve conserved genes was constructed for representative published strains alongside *C. jejuni* 1336 and *C. jejuni* 414. For each of the 31 strains considered, the 12 conserved genes were identified (initiation factor 2, InfB; elongation factor G, FusA; elongation factor Tu, Tuf; ribosomal protein L2, RplB; ribosomal protein S5, RpsE; ribosomal protein S8, RpsH; ribosomal protein S11, RpsK; DNA topoisomerase I, TopA; signal recognition particle protein, Ffh; DNA gyrase B subunit, GyrB; GTP-binding protein, LepA; CTP synthase, PyrG).

Nucleotide sequences or translations of the twelve genes were aligned with the computer program MUSCLE v3.6 (<http://www.drive5.com/muscle/>) and the alignments were edited to remove gaps and merged into single alignment. A maximum likelihood phylogeny was then prepared using the software dnaml (for nucleotide sequences) or proml (for predicted protein sequences), part of the PHYLIP software package (version 3.67).

**Ortholog dendogram and construction of Venn diagrams**

Orthologous genes among genomes were identified with OrthoMCL v1.4

([http://orthomcl.org/cgi-bin/OrthoMclWeb.cgi?rm=orthomcl#Software](http://orthomcl.org/cgi-bin/OrthoMclWeb.cgi?rm=orthomcl#Software)) using default settings. A pairwise comparison of each genome sequence was undertaken, calculating the average ortholog frequency between each genome pair. From these data a distance matrix was constructed and clustered with the neighbour joining software neighbor, also part of PHYLIP.

The ORFs included in the analysis depicted in Figure 3 represent the number of orthologous genes within *C. jejuni* strains NCTC 11168, 1336 and 414 as defined by the OrthoMCL algorithm which was used to bin the ORFs. Hence, although 1643 ORFs are present in the current NCTC11168 genome annotation, 1607 are represented on the figure. This avoids the ambiguity introduced by possible paralogs.

Reference List

Carver,T.J., Rutherford,K.M., Berriman,M., Rajandream,M.A., Barrell,B.G., and Parkhill,J. (2005) ACT: the Artemis Comparison Tool. *Bioinformatics* **21:** 3422-3423.

Champion,O.L., Gaunt,M.W., Gundogdu,O., Elmi,A., Witney,A.A., Hinds,J. et al. (2005) Comparative phylogenomics of the food-borne pathogen Campylobacter jejuni reveals genetic markers predictive of infection source. *Proc Natl Acad Sci U S A* **102:** 16043-16048.

Fouts,D.E., Mongodin,E.F., Mandrell,R.E., Miller,W.G., Rasko,D.A., Ravel,J. et al. (2005) Major structural differences and novel potential virulence mechanisms from the genomes of multiple campylobacter species. *PLoS Biol* **3:** e15.

Kim,C.C., Joyce,E.A., Chan,K., and Falkow,S. (2002) Improved analytical methods for microarray-based genome-composition analysis. *Genome Biol* **3:** RESEARCH0065.

Rutherford,K., Parkhill,J., Crook,J., Horsnell,T., Rice,P., Rajandream,M.A., and Barrell,B. (2000) Artemis: sequence visualization and annotation. *Bioinformatics* **16:** 944-945.
