# Supplementary figures and images for: Genomic variations define divergence of water/wildlife-associated Campylobacter jejuni niche specialists from common clonal complexes
Source: Environ Microbiol. 2011 Mar 21;13(6):1549–60. doi: 10.1111/j.1462-2920.2011.02461.x (PMC3569610; doi:10.1111/j.1462-2920.2011.02461.x)

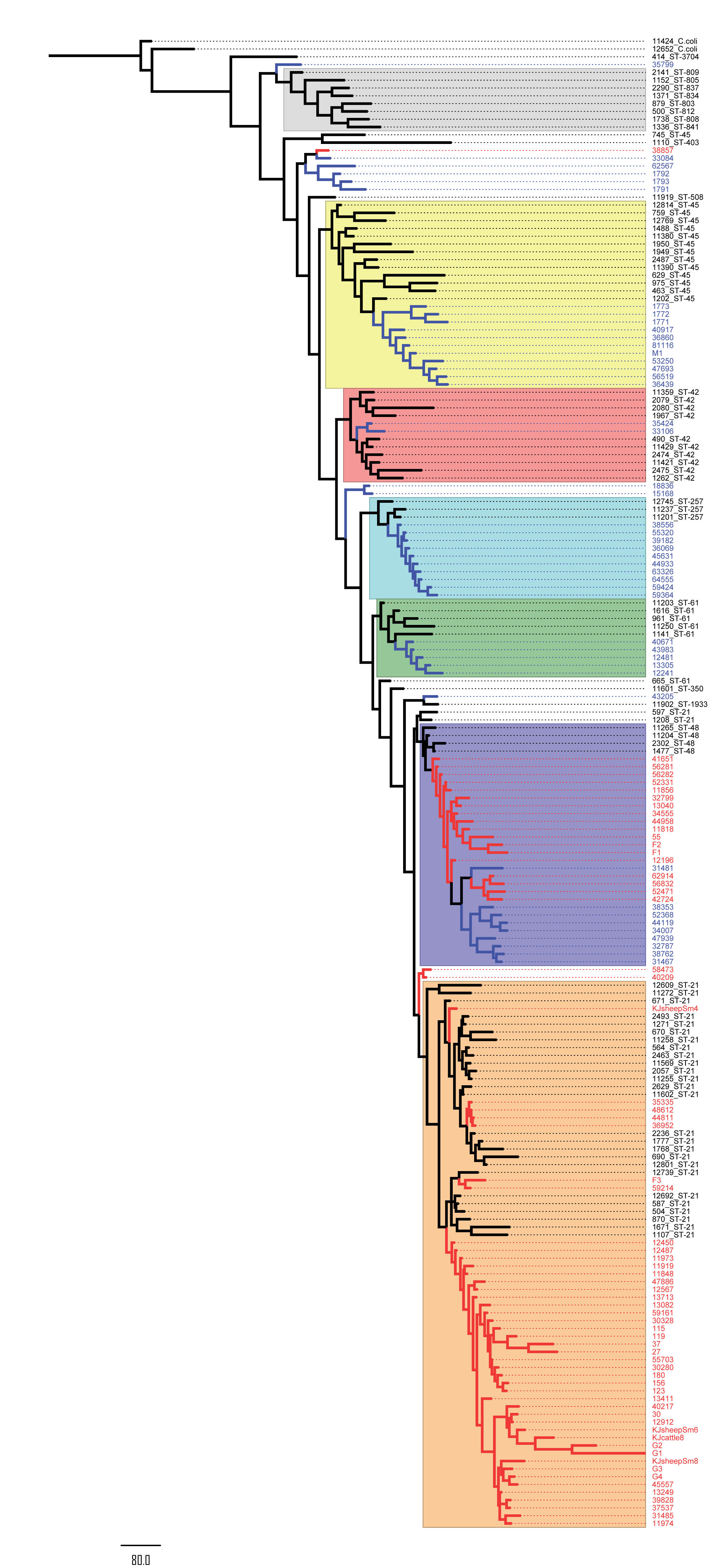

Supplement: Fig S2 — Dendrogram of Campylobacter and related strains based on concatenated nucleotide sequences. [file emi0013-1549-sd2.tif]

0.1

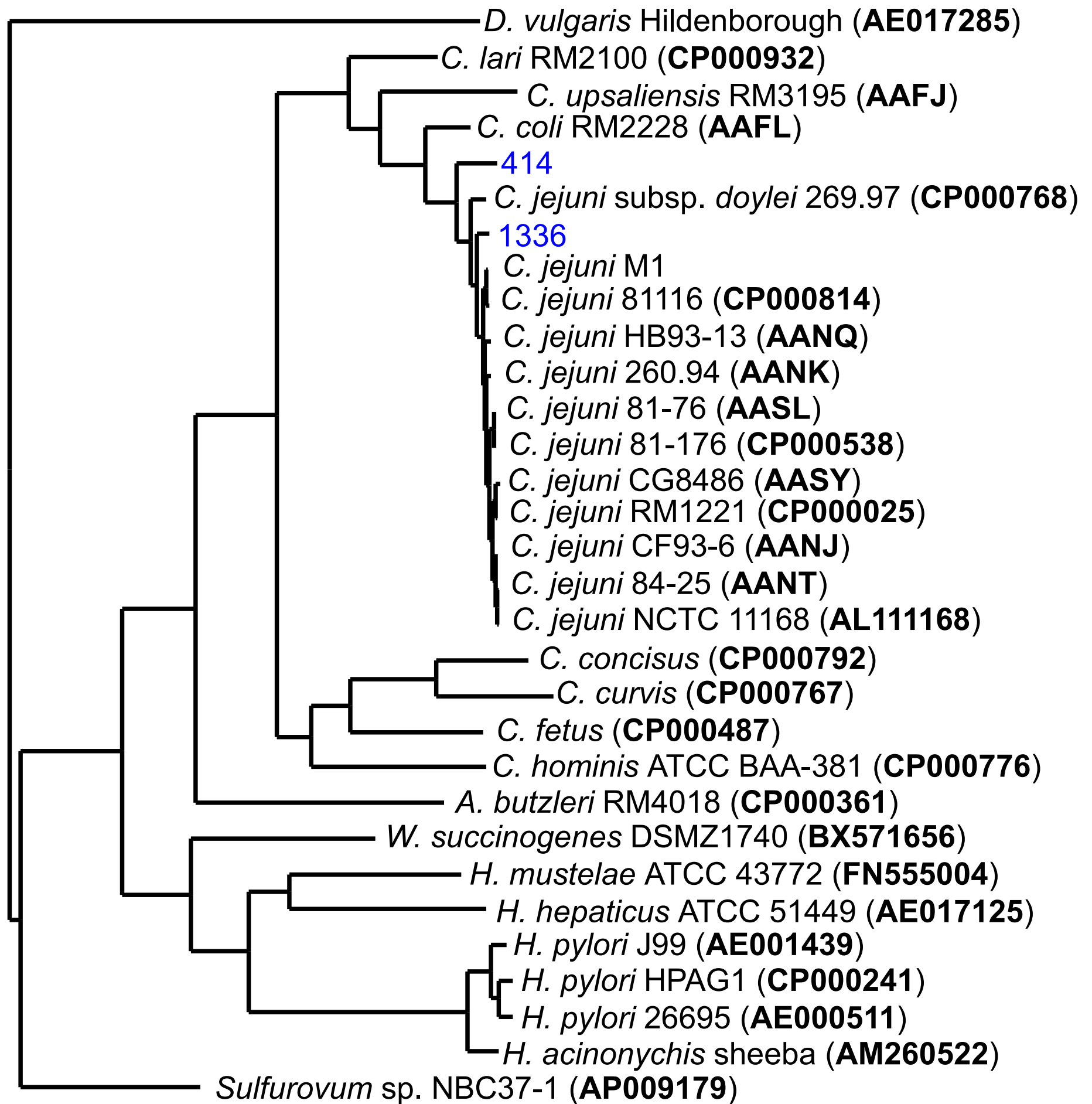

Supplement: Fig S3 — Dendrogram of Campylobacter and related strains based on predicted protein orthologues. [file emi0013-1549-sd3.pdf]

0.01

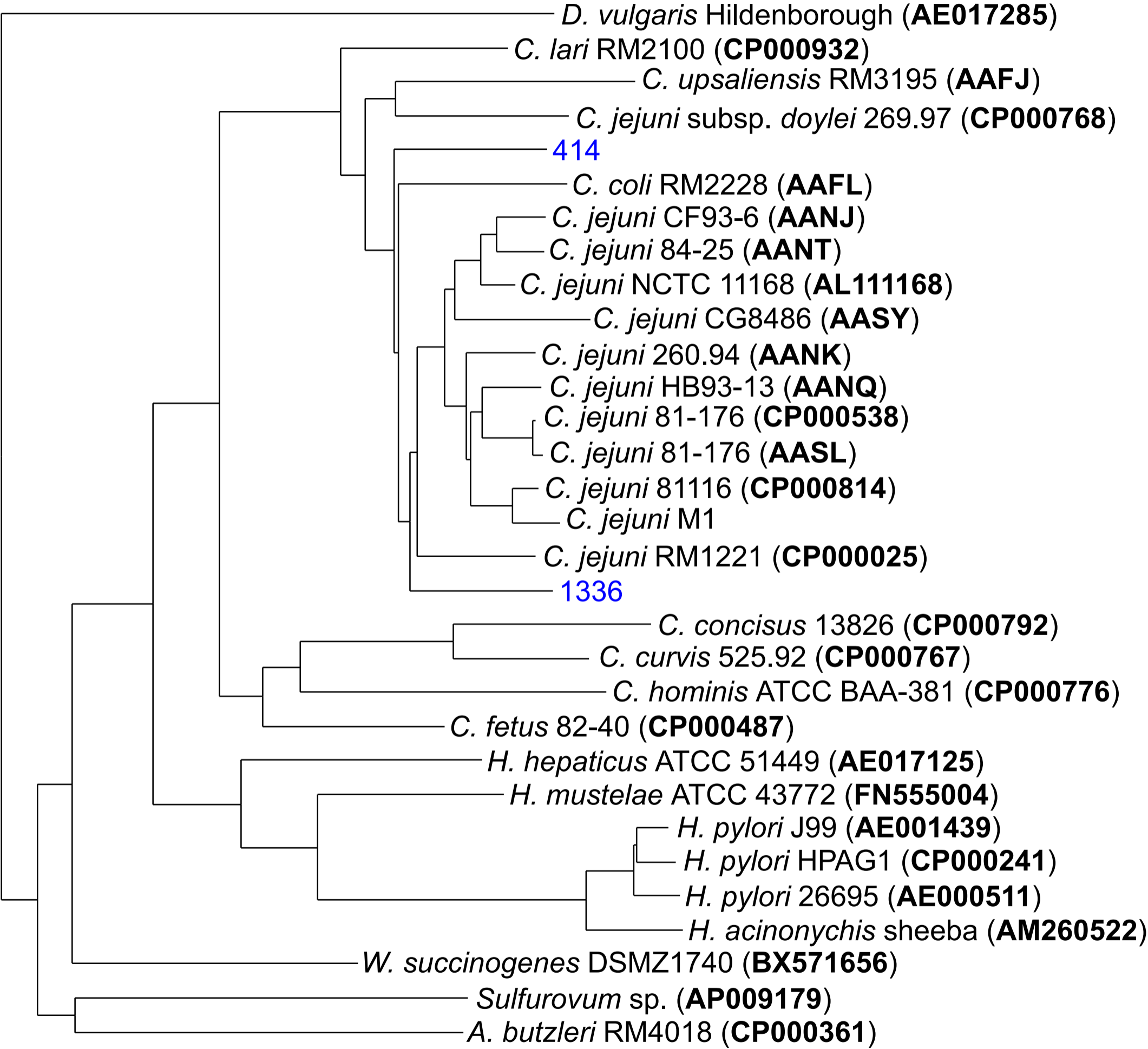

Supplement: Table S1 — Summary of microarray comparative genome hybridizations (CGH) to indicate the presence or absence/divergence of selected genomic loci and distribution among the strains. [file emi0013-1549-sd4.pdf]
