# Supplementary material for: Genomic variations define divergence of water/wildlife-associated Campylobacter jejuni niche specialists from common clonal complexes
Source: Environ Microbiol. 2011 Mar 21;13(6):1549–60. doi: 10.1111/j.1462-2920.2011.02461.x (PMC3569610; doi:10.1111/j.1462-2920.2011.02461.x)
Supplement: Table S3 — Summary of genome sequence data. [file emi0013-1549-sd6.doc]

Table S2. Regions of divergence associated with the WW / ST-3704 isolates according to CGH

| Region | Reference genome | ORFs | Comments* |
| --- | --- | --- | --- |
|  |  |  |  |
| PH01 | NCTC11168 | CJ0077c-0079c | Cytolethal distending toxin genes (*cdtABC*) |
| PH02 | NCTC11168 | CJ0201c-0202c | Includes membrane protein |
| PH03 | RM1221 | CJE0202-0212 | Includes pathogenicity-related proteins |
| PH04a | NCTC11168 | CJ0289c-0290c | Includes *peb3*, *glpT*; PR1, Tab4, Par4 |
| PH04b | NCTC11168 | CJ0295-0300c | Includes *panBCD*, *modC*; PR1, Tab4, Par4 |
| PH05 | 81176 | CJJ81176_0523-0530 | Periplasmic / membrane proteins |
| PH06 | NCTC11168 | CJ0548-0549 | *fliD*, *fliS* |
| PH07 | NCTC11168 | CJ0553-0554 | Includes membrane protein |
| PH08 | NCTC11168 | CJ0563-0564 | Includes membrane protein; Tab7, Par7 |
| PH09 | NCTC11168 | CJ0570-0571 | Includes transcriptional regulator; Tab7, Par7 |
| PH10a | NCTC11168 | CJ0727-0737 | Includes ABC transporters; PR3, Tab9, Par9 |
| PH10b | RM1221 | CJE0839-0843 | Includes membrane protein; Tab9, Par9 |
| PH11 | NCTC11168 | CJ0968-0975 | Includes periplasmic / membrane proteins; Tab10, Par10 |
| PH12 | RM1221 | CJE1093-1154 | CJIE3-like phage |
| PH13 | NCTC11168 | CJ1198-1203c | Includes *luxS*, *metE*, *metF* |
| PH14 | NCTC11168 | CJ1541-1564 | Includes transcriptional regulators; Tab14, Par14 |
| PH15^†^ | NA | SSH-04 | Periplasmic protein |
|  |  |  |  |

*Variable regions as identified previously by (Pearson et al., 2003) (PR), (Dorrell et al., 2001) (Dor), (Parker et al., 2006) (Par), (Taboada et al., 2004) (Tab), or our CGH data (PH; regions of divergence associated with the WW group of isolates)

^†^ NA; Not applicable: SSH-4 is a subtracted sequence (Hepworth et al., 2007)

Reference List

Dorrell,N., Mangan,J.A., Laing,K.G., Hinds,J., Linton,D., Al Ghusein,H. et al. (2001) Whole genome comparison of Campylobacter jejuni human isolates using a low-cost microarray reveals extensive genetic diversity. *Genome Res* **11:** 1706-1715.

Hepworth,P.J., Leatherbarrow,H., Hart,C.A., and Winstanley,C. (2007) Use of suppression subtractive hybridisation to extend our knowledge of genome diversity in Campylobacter jejuni. *BMC Genomics* **8:** 110.

Parker,C.T., Quinones,B., Miller,W.G., Horn,S.T., and Mandrell,R.E. (2006) Comparative genomic analysis of Campylobacter jejuni strains reveals diversity due to genomic elements similar to those present in C. jejuni strain RM1221. *J Clin Microbiol* **44:** 4125-4135.

Pearson,B.M., Pin,C., Wright,J., I'Anson,K., Humphrey,T., and Wells,J.M. (2003) Comparative genome analysis of Campylobacter jejuni using whole genome DNA microarrays. *FEBS Lett* **554:** 224-230.

Taboada,E.N., Acedillo,R.R., Carrillo,C.D., Findlay,W.A., Medeiros,D.T., Mykytczuk,O.L. et al. (2004) Large-scale comparative genomics meta-analysis of Campylobacter jejuni isolates reveals low level of genome plasticity. *J Clin Microbiol* **42:** 4566-4576.
