# Supplementary material for: Genomic variations define divergence of water/wildlife-associated Campylobacter jejuni niche specialists from common clonal complexes
Source: Environ Microbiol. 2011 Mar 21;13(6):1549–60. doi: 10.1111/j.1462-2920.2011.02461.x (PMC3569610; doi:10.1111/j.1462-2920.2011.02461.x)
Supplement: Table S4 — Novel features of the C.jejuni 1336 and 414 genomes in comparison with NCTC11168. The data presented are based on comparisons of genome sequence data. [file emi0013-1549-sd7.doc]

Table S3. Summary of genome sequence data.

|  | 1336 | 414 |
| --- | --- | --- |
|  |  |  |
| Scaffolds: |  |  |
| Length of coverage (bp) | 1,702,233 | 1,709,525 |
| Number of scaffolds | 6 | 6 |
| Depth of coverage | 21.65X | 18.60X |
|  |  |  |
| Contigs: |  |  |
| Length of coverage (bp) | 1,697,564 | 1,700,255 |
| Number of contigs | 89 | 71 |
| Depth of coverage | 21.77X | 18.71X |
| Number included in scaffolds | 35 | 35 |
|  |  |  |
| Reads: |  |  |
| Number of reads | 245,184 | 196,469 |
| Coverage (bp) | 38,044,631 | 32,164,697 |
| Number of aligned reads | 237,473 | 195,474 |
| Coverage (bp) | 36,860,495 | 31,758,867 |
|  |  |  |
|  |  |  |
