# Supplementary material for: Genomic variations define divergence of water/wildlife-associated Campylobacter jejuni niche specialists from common clonal complexes
Source: Environ Microbiol. 2011 Mar 21;13(6):1549–60. doi: 10.1111/j.1462-2920.2011.02461.x (PMC3569610; doi:10.1111/j.1462-2920.2011.02461.x)
Supplement: Table S5 — Selected novel genomic regions of strains 1336 and 414 targeted for PCR assays. [file emi0013-1549-sd8.doc]

**Table S4.** Novel features of the *C. jeuni* 1336 and 414 genomes in comparison with NCTC11168. The data presented are based on comparisons of genome sequence data.

| **NCTC11168 ORF** | **INS / DEL / DIV*** | **Comment on putative functions** | **VR†** |
| --- | --- | --- | --- |
|  |  |  |  |
| Cj0006 | DEL from 414 | Na^+^/H^+^ antiporter family protein |  |
| Cj0008 | DEL from 414 | Hypothetical protein |  |
| Cj0021 | DEL from 414 | Putative fumarylacetoacetate hydrolase family protein |  |
| Cj0030-33 | DIV in both | Restriction-modification system and outer membrane protein / 1336: Includes gamma-glutamyltransferase, membrane proteins, hypothetical proteins, cytochrome C biogenesis protein (matching other *C. jejuni*) / 414: Includes gamma-glutamyltransferase, integral membrane protein (matching other *C. jejuni*) and hypothetical proteins (non-*Campylobacter* matches); Cj0030 and Cj0031 also deleted from 81-176 (Hofreuter et al., 2006) | Tab1  Par1 |
| Cj0046 | DIV in both | Pseudogene in NCTC11168 (transport-related) / full gene in 1336 / mostly deleted from 414 |  |
| Cj0055-58 | DEL / DIV | Hypothetical proteins and putative peptidase (Cj0058) / 1336: only Cj0055 and Cj0056 are divergent / 414: All four deleted. Cj0057 reported as down-regulated during chick colonization (Woodall et al., 2005) | Tab2  Par2 |
| Cj0071-2 | DEL from 414 | Pseudogene (putative iron-binding protein) |  |
| Cj0077-79 | DEL from both | Cytolethal distending toxin A,B,C / 1336: only *cdtA* deleted, *cdtB* and *cdtC* present but pseudogenes / 414: all deleted | PH01 |
| Cj0092-93 | DEL from 414; DIV from 1336 | Putative periplasmic proteins / 1336: only Cj0092 is divergent / 414: both deleted |  |
| Cj0122 | DIV in 1336 | Hypothetical protein |  |
| (Cj0121-123) | INS in 414 | 8.6 kb insertion. Glycerol-3-phosphate cytidylyltransferase, hypothetical proteins, CDP glycerol glycerophosphotransferase |  |
| Cj0139-140 | DIV from both | Putative endonuclease and hypothetical protein / 1336: DNA (Cytosine-5-)-methyltransferase and hypothetical protein / 414: Restriction endonuclease and hypothetical proteins |  |
| Cj0177-181 | DEL from both | Iron transport proteins, including TonB protein and receptor; Cj0178 mutant shows reduced colonization in a chick model (Palyada et al., 2004); Cj0177 - Cj0181 also deleted from 81-176 (Hofreuter et al., 2006) | Tab3  Par3 |
| Cj0197-198 | INS in 414 | Sodium:sulfate symporter family protein |  |
| Cj0201-202 | DIV in 1336 | Integral membrane protein and hypothetical protein / 1336: Cj0202 is deleted | PH02 |
| Cj0207-208 | INS in 414 | Pseudogene; restriction endonuclease (no *Campylobacter* matches) |  |
| Cj0223 | DEL from both | Pseudogene in 11168 (putative IgA protease family protein) | PH03 |
| Cj0241 | DIV in 414 | Putative iron-binding protein; multiple pseudogene in 414 |  |
| Cj0250 | DEL from 1336 | Putative Major Facilitator Superfamily transport protein |  |
| Cj0264-265 | DEL from 414 | Molybdopterin containing oxidoreductase and putative cytochrome C-type haem-binding periplasmic protein |  |
| Cj0288-300 | INS / DIV in both | Large insertion / divergent region, contains *peb3* (deleted from 1336 only) and *panBCD* (deleted from both 1336 and 414) / 1336: insertion includes *cdtABC*-like genes (57-80% identity with *C. lari* proteins; C1336_00006_27-29), restriction modification proteins, adenine-specific DNA methylase, class D beta-lactamase, acid phosphatase / 414: insertion includes restriction modification proteins, adenine-specific DNA methylase, putative ABC transporter subunits, phage Gp37Gp68 family protein. Cj0297 and Cj0298 (*panB*, *panC*) reported as down-regulated during chick colonization (Woodall et al., 2005); Cj0299 deleted from 81-176 (Hofreuter et al., 2006) | PR1  Tab4  Par4  PH04 |
| Cj0339-340 | DIV/ DEL in 414 | Major facilitator superfamily protein and putative nucleoside hydrolase / 414: Cj0339 deleted |  |
| Cj0380 | DEL from both | Hypothetical protein; also deleted from 81-176 (Hofreuter et al., 2006) |  |
| Cj0414-415 | DIV in 414 | Oxidoreductase sub-units / 414: matches to hypothetical proteins; Cj0414/0415 reported as down-regulated during chick colonization (Woodall et al., 2005); Cj0414 required for chicken colonisation (Pajaniappan et al., 2008) |  |
| (Cj0415-417) | INS in 1336 | 1336: Hypothetical protein |  |
| Cj0423-425 | DIV in both | Putative integral membrane protein, putative acidic periplasmic protein and putative periplasmic protein / 1336: includes putative lipoprotein, membrane protein, hypothetical proteins (matching other *Campylobacter* proteins) / 414: partial matches to putative lipoprotein, membrane protein, hypothetical proteins (matching other *Campylobacter* proteins). Cj0425 reported as down-regulated during chick colonization (Woodall et al., 2005) | Tab5  Par5 |
| (Cj0429-430) | INS in 414 | Includes regulatory protein GntR, HTH:GntR, transporter protein, tricarballylate dehydrogenase, putative citrate utilization protein B |  |
| Cj0437-439 | DIV in 414 | Succinate dehydrogenase flavoprotein (subunit A and B divergent, subunit C deleted, but not a complete deletion); Cj0437-0439 reported as up-regulated during chick colonization (Woodall et al., 2005) |  |
| Cj0453 | DIV in 414 | Thiamin biosynthesis protein ThiC; reported as down-regulated during chick colonization (Woodall et al., 2005) |  |
| Cj0480-490 | DEL from 414 | Transcriptional regulator, dihydrodipicolinate synthase, altronate hydrolase, MFS transport protein, oxidoreductase, sugar transporter, amidohydrolase and putative aldehyde dehydrogenase; Cj0484, Cj0487, Cj0488 and Cj0489 also deleted from 81-176 (Hofreuter et al., 2006) | PR2  Tab6  Par6 |
| (Cj0481-482) | INS in 1336 | Putative 2-keto-3-deoxygluconate transporter (matching non-*Campylobacter* proteins) |  |
| Cj0501 | DEL from 414 | Pseudogene (ammonium transporter) |  |
| (Cj0508-509) | INS in 414 | Includes matches to *Campylobacter* phage tail protein, phage cI-like repressor, hypothetical protein (matching non-*Campylobacter* proteins), putative sugar transferase (414 Region 3) |  |
| Cj0517 | DEL from 414 | CrcB protein homolog (membrane protein), only partially present in 414 |  |
| Cj522-523 | DIV in 414 | Na+/Pi cotransporter protein and putative membrane protein. Present but divergent and pseudogene in 414 |  |
| (Cj0538-539) | INS in 414 | Hypothetical protein (matches *Helicobacter*) and putative glycosyl transferase (62% identity with *Campylobacter*) |  |
| Cj0548 | DIV in 1336 | FliD, flagellar hook protein (74% identity); mutant enhanced in rabbit ileal colonization (Stintzi et al., 2005) | PH06 |
| Cj0552-554 | DEL from both | Two putative membrane proteins and a hypothetical protein | PH07 |
| Cj0563-571 | DEL from both | Putative integral membrane protein, putative ATP/GTP binding protein, putative transcriptional regulator and hypotheticals; Cj0571 mutant unaffected in colonization of rabbit ileal loop model (Stintzi et al., 2005); Integration hot spot (Hofreuter et al., 2006) | Tab7  Par7  PH08 |
| Cj0617-618 | DEL from 1336 | Hypothetical proteins; Cj618 mutant attenuated for chick colonization (Hendrixson and DiRita, 2004) |  |
| Cj0628 | DEL/DIV in 414 | Putative lipoprotein / 414: Partial match to *Campylobacter* restriction enzyme; Cj0628 reported as down-regulated during chick colonization (Woodall et al., 2005) | Tab8  Par8 |
| (Cj0653-659) | INS in both | 1336: insert is di-/tripeptide transporter / 414: much larger insert includes di-/tripeptide transporter, bacteriophage proteins (CMLP1-like element), putative type VI secretion proteins (414 Region 1) |  |
| Cj0672-679 | DEL from 414 | Putative periplasmic protein, pseudogene (potassium-transporting ATPase A chain, potassium-transporting ATPase B chain, pseudogene C chain and truncated D protein); *kdpABC* are putatively functional in strain 81-176 (Hofreuter et al., 2006) |  |
| (Cj0684-685) | INS in 414 | Pyruvate kinase , sugar-1-phosphate nucleotidyltransferase, methyltransferase, sugar nucleotidyltransferase (matching *Campylobacter* proteins) |  |
| Cj0685-687 | DIV in 414 | Includes insertion of putative O-acetyl transferase (matching non-*Campylobacter* protein) and adenylylsulfate kinase |  |
| Cj0690 | DEL from both | Putative restriction/modification enzyme |  |
| Cj0727-755 | DIV / DEL in both | Major divergent / deleted region in both includes deletions of putative periplasmic solute-binding protein, putative type I phosphodiesterase/nucleotide pyrophosphatase, ABC transport system proteins, putative HAD-superfamily hydrolase, putative haemagglutination activity domain, rRNA locus, transposase (pseudogene), TonB transport protein, ferric enterobactin uptake receptor CfrA (Cj0755) / 1336: Cj0727-Cj0755 deleted, replacement includes hypothetical protein matching other *C. jejuni* and putative lipoprotein but pseudogenome contains sequence gap in this region/ 414: Cj0735-Cj0755 is deleted; Cj0753 also deleted from 81-176 (Hofreuter et al., 2006) | PR3  Tab9  Par9  PH10 |
| (Cj0759-760) | INS in 414 | Includes restriction-modification proteins (matches to both *Campylobacter* and non-*Campylobacter* proteins) |  |
| Cj0786 | DIV in 414 | 11168: Small hydrophobic protein; 414: transporter, LysE family (*C. coli* match) and partial match to Cj0786 |  |
| Cj0794 | DEL / DIV in both | Hypothetical protein / 1336: divergent / 414: deleted |  |
| Cj0814-816 | DIV/DEL in both | Hypothetical proteins / 1336: replaced by a larger region including hypothetical proteins, bacteriocin resistance protein (matching *Campylobacter* proteins), nickase (pTet plasmid) / 414: deleted |  |
| Cj0818 | DEL from both | Putative lipoprotein |  |
| Cj0830 | DEL from 414 | Putative integral membrane protein; reported as down-regulated during chick colonization (Woodall et al., 2005) |  |
| Cj0859-0860 | DIV in both | 11168: Putative integral membrane protein and hypothetical protein / 1336: shares similarity with putative toxin-like outer membrane protein (autotransporter domain) / 414 some similarity with 1336, but pseudogene; Cj0859 (FspA) implicated in virulence (Poly et al., 2007) |  |
| Cj0864 | DIV in both | Putative periplasmic protein. 414: part match to thiol:disulfide interchange protein DsbA; reported as up-regulated during chick colonization (Woodall et al., 2005) |  |
| Cj0866 | DEL from 414 | Pseudogene (arylsulfatase) / 1336: full gene present / 414: deleted; reported as up-regulated during chick colonization (Woodall et al. 2005); putatively functional in strain 81-176 (Hofreuter et al., 2006) |  |
| Cj0873-876 | DEL from 414 | Hypothetical protein, putative cytochrome C and putative periplasmic protein; Cj0874 and Cj0876 reported as down-regulated during chick colonization (Woodall et al., 2005) |  |
| (Cj0887-888) | INS in 1336 | Contains matches to CJIE3-like prophage element, including phage integrase, also includes region with ORFs matching *Helicobacter* sp. proteins, mostly low identity matches, Vgr proteins, UV damage repair protein and hypothetical proteins matching *Campylobacter* proteins (1336 Regions 1A and 1B; relative positions unconfirmed) |  |
| Cj0903 | DEL from 414 | Putative amino-acid transport protein; mutation attenuated for chick colonization (Hendrixson and DiRita, 2004) |  |
| Cj0908 | DIV in both | Putative periplasmic protein, partial matches in both |  |
| (Cj0936-937) | INS in 1336 | CJIE2-like prophage, but divergent (1336 Region 2) |  |
| Cj0937 | DIV in 414 | Putative integral membrane protein |  |
| Cj0967-975 | DEL from both | Putative periplasmic proteins, hypothetical proteins and a putative integral membrane protein | Tab10  Par10  PH11 |
| (Cj0983-984) | INS in both | Putative metal-dependent hydrolase / sulphatase. Similar in both but pseudogene in 414 (1336 Region 3) |  |
| Cj0987-990 | DIV in both | Putative MFS transport protein, putative membrane protein and hypotheticals; Cj0987 reported as down-regulated during chick colonization (Woodall et al., 2005) |  |
| Cj1007 | DIV in 414 | Putative mechanosensitive ion channel family protein |  |
| (Cj1030-1031) | INS in 414 | Transporters, siderophore-related and siderophore receptor |  |
| (Cj1046-1047) | INS in 414 | Includes hypothetical protein, replication-linked phage protein, restriction-modification proteins |  |
| Cj1055 | DIV in 414 | Putative integral membrane protein (sulfatase domain) |  |
| Cj1063 | DEL from 414 | Putative acetyltransferase |  |
| (Cj1069-1070) | INS in both | 1336: Transporter, MFS superfamily and hypothetical protein / 414: probable pyridine nucleotide-disulfide oxidoreductase YkgC and domain of unknown function superfamily protein |  |
| Cj1122 | DEL from both | Putative integral membrane protein |  |
| Cj1135-1149 | DIV from both | LOS locus (Cj1131-1152); different lengths of replacement regions, larger in 1336 (1336 Region 4) | Dor3  PR4  Tab11  Par11 |
| Cj1158-1160 | DIV in both | Small hydrophobic proteins and putative membrane protein. Same inserted ORF in both 1336 and 414: twin-arginine translocation pathway signal protein matching other *Campylobacter* proteins |  |
| Cj1167 | DEL from both | L-lactate dehydrogenase (ldh) |  |
| Cj1183 | DEL from 414 | Cyclopropane-fatty-acyl-phospholipid synthase; reported as down-regulated during chick colonization (Woodall et al., 2005) |  |
| Cj1187 | DEL from 414 | Arsenical pump membrane protein (partial deletion) |  |
| Cj1198-1202 | DEL from 1336 | S-ribosylhomocysteine lyase (autoinducer-2, LuxS), putative iron/ascorbate-dependent oxidoreductase, putative NLPA family lipoprotein, 5-methyltetrahydropteroyltriglutamate homocysteine methyltransferase and 5,10-methylenetetrahydrofolate reductase | PH13 |
| (Cj1221-1222) | INS in 414 | Putative sodium/pantothenate symporter and hypothetical protein (both matching *C. coli*) |  |
| Cj1237 | DIV in 414 | Putative phosphatase |  |
| Cj1240 | DEL from 414 | Putative periplasmic protein; pseudogene in 1336 |  |
| Cj1255 | DEL from 1336 | Putative isomerase |  |
| (Cj1282-1283) | INS in 414 | CJIE4-like element but with some regions of divergence |  |
| Cj1297 | DEL from both | Hypothetical protein |  |
| Cj1309-1310 | DIV in 1336 | Hypothetical proteins |  |
| (Cj1313-1314) | INS in 414 | Acetyl transferase |  |
| Cj1305-1341 | DIV in both | Glycosylation locus, flagellins, motility accessory factors; Cj1321-1325 identified by Champion et al. (Champion et al., 2005) as characteristic to livestock isolates (1336 Region 5) | Dor2  PR5  Tab12  Par12 |
| (Cj1355-1356) | INS in 414 | Hypothetical proteins |  |
| (Cj1359-1360) | INS in 1336 | Restriction-modification proteins (1336 Region 6) |  |
| Cj1365 | DEL from 1336 | Putative secreted serine protease. Identified as lacking from the non-livestock clade of Champion et al. (Champion et al., 2005) |  |
| Cj1395-1398 | DIV in 414 | Pseudogene (putative MmgE/PrpD family protein), putative ferrous iron transport proteins; Cj1395 is putatively functional in strain 81-176 (Hofreuter et al., 2006) |  |
| Cj1406 | DEL from 414 | Putative periplasmic protein |  |
| Cj1415-1442 | DIV / DEL in both | Capsule locus; differences between 1336 and 414 (1336 Region 7) | Dor1  PR6  Tab13  Par13 |
| (Cj1454-1455) | INS in 414 | Two putative sugar transferases |  |
| Cj1456 | DEL from 414 | Putative periplasmic protein |  |
| (Cj1461-1462) | INS in 1336 | Putative sugar transferase |  |
| (Cj1520-1521) | INS in 414 | Includes putative mobilisation protein (low identity match with *C. coli* pCC2228-2) and putative KAP family P-loop domain protein |  |
| Cj1541-1564 | DIV in both | 1336: includes restriction modification proteins and proteins with low identity matches to *Rhodococcus* proteins, putative aldo/keto reductase (matching *Rhodoferax ferrireducens* T118). / 414: Divergent region is different; includes major facilitator family transporter, flavodoxin-like protein, putative permease, aldo/keto reductase (matching *Rhodoferax ferrireducens* T118), hypothetical protein (matching *Helicobacter hepaticus*) (1336 Region 8; 414 Region 2) | Tab14  Par14  PH14 |
| Cj1585 | DIV in 1336 | Putative oxidoreductase, also deleted from 81-176 (Hofreuter et al., 2006); 1336: includes anaerobic dimethyl sulfoxide reductase chain A matches but is pseudogene |  |
| (Cj1599-1600) | INS in 414 | Hypothetical protein and type II modification methylase (both matching *Helicobacter* proteins) |  |
| Cj1602 | DEL from 1336 | Hypothetical protein |  |
| (Cj1620-1621) | INS in 1336 | Hypothetical protein (matching *C.coli*) and putative methyl-accepting chemotaxis protein |  |
| Cj1627 | DEL from 414 | Hypothetical protein |  |
| Cj1631 | DIV in 414 | Conserved hypothetical protein |  |
| (Cj1633-1634) | INS in 414 | Haemolysin secretion/activation protein and large haemagglutination activity domain protein (FHA-like, matching *C. fetus*) |  |
| Cj1667-1668 | DIV in both | RepA protein homolog and putative periplasmic protein; Cj1668 reported as down-regulated during chick colonization (Woodall et al., 2005) |  |
| Cj1677-1679 | DEL from both | Putative lipoprotein. 1336: only Cj1677 and Cj1678 are deleted | Tab15  Par15 |
| (Cj1714-1715) | INS in both | LPS core biosynthesis protein matching RM1221 CJE1884 (94% identity); same insertion in both 1336 and 414 |  |
| Cj1717-1727 | DEL/ DIV in both | Putative periplasmic proteins, homoserine O-succinyltransferase and putative O-acetylhomoserine (thiol)-lyase deleted from both; 1336: 3-isopropylmalate dehydratase large subunit, 3-isopropylmalate dehydrogenase, 2-isopropylmalate synthase (LeuCBA) are also deleted; Cj01725 (deleted from both) reported as down-regulated during chick colonization (Woodall et al., 2005) | PR7  Tab16  Par16 |
|  |  |  |  |

* INS / DEL indicates insertion or deletion with reference to the NCTC11168 genome. DIV; divergent. In the case of a deletion, the equivalent NCTC11168 ORFs flanking the deletion are indicated in parentheses.

^†^ Variable regions as identified previously by (Pearson et al., 2003) (PR), (Dorrell et al., 2001) (Dor), (Parker et al., 2006) (Par), (Taboada et al., 2004) (Tab), or our CGH data (PH; regions of divergence associated with the WW group of isolates)

Reference List

Champion,O.L., Gaunt,M.W., Gundogdu,O., Elmi,A., Witney,A.A., Hinds,J. et al. (2005) Comparative phylogenomics of the food-borne pathogen Campylobacter jejuni reveals genetic markers predictive of infection source. *Proc Natl Acad Sci U S A* **102:** 16043-16048.

Dorrell,N., Mangan,J.A., Laing,K.G., Hinds,J., Linton,D., Al Ghusein,H. et al. (2001) Whole genome comparison of Campylobacter jejuni human isolates using a low-cost microarray reveals extensive genetic diversity. *Genome Res* **11:** 1706-1715.

Hendrixson,D.R. and DiRita,V.J. (2004) Identification of Campylobacter jejuni genes involved in commensal colonization of the chick gastrointestinal tract. *Mol Microbiol* **52:** 471-484.

Hofreuter,D., Tsai,J., Watson,R.O., Novik,V., Altman,B., Benitez,M. et al. (2006) Unique features of a highly pathogenic Campylobacter jejuni strain. *Infect Immun* **74:** 4694-4707.

Pajaniappan,M., Hall,J.E., Cawthraw,S.A., Newell,D.G., Gaynor,E.C., Fields,J.A. et al. (2008) A temperature-regulated Campylobacter jejuni gluconate dehydrogenase is involved in respiration-dependent energy conservation and chicken colonization. *Mol Microbiol* **68:** 474-491.

Palyada,K., Threadgill,D., and Stintzi,A. (2004) Iron acquisition and regulation in Campylobacter jejuni. *J Bacteriol* **186:** 4714-4729.

Parker,C.T., Quinones,B., Miller,W.G., Horn,S.T., and Mandrell,R.E. (2006) Comparative genomic analysis of Campylobacter jejuni strains reveals diversity due to genomic elements similar to those present in C. jejuni strain RM1221. *J Clin Microbiol* **44:** 4125-4135.

Pearson,B.M., Pin,C., Wright,J., I'Anson,K., Humphrey,T., and Wells,J.M. (2003) Comparative genome analysis of Campylobacter jejuni using whole genome DNA microarrays. *FEBS Lett* **554:** 224-230.

Poly,F., Ewing,C., Goon,S., Hickey,T.E., Rockabrand,D., Majam,G. et al. (2007) Heterogeneity of a Campylobacter jejuni protein that is secreted through the flagellar filament. *Infect Immun* **75:** 3859-3867.

Stintzi,A., Marlow,D., Palyada,K., Naikare,H., Panciera,R., Whitworth,L., and Clarke,C. (2005) Use of genome-wide expression profiling and mutagenesis to study the intestinal lifestyle of Campylobacter jejuni. *Infect Immun* **73:** 1797-1810.

Taboada,E.N., Acedillo,R.R., Carrillo,C.D., Findlay,W.A., Medeiros,D.T., Mykytczuk,O.L. et al. (2004) Large-scale comparative genomics meta-analysis of Campylobacter jejuni isolates reveals low level of genome plasticity. *J Clin Microbiol* **42:** 4566-4576.

Woodall,C.A., Jones,M.A., Barrow,P.A., Hinds,J., Marsden,G.L., Kelly,D.J. et al. (2005) Campylobacter jejuni gene expression in the chick cecum: evidence for adaptation to a low-oxygen environment. *Infect Immun* **73:** 5278-5285.
