# Supplementary material for: Genomic variations define divergence of water/wildlife-associated Campylobacter jejuni niche specialists from common clonal complexes
Source: Environ Microbiol. 2011 Mar 21;13(6):1549–60. doi: 10.1111/j.1462-2920.2011.02461.x (PMC3569610; doi:10.1111/j.1462-2920.2011.02461.x)
Supplement: Table S6 — Details of oligonucleotide primers for PCR assays. [file emi0013-1549-sd9.doc]

**Table S5.** Selected novel genomic regions of strains 1336 and 414 targeted for PCR assays

| **Region designation** | **Size (kb; approx)** | **Location relative to NCTC11168 genome** | **Description / database matches** |
| --- | --- | --- | --- |
|  |  |  |  |
| 1336-Region 1A* | >38 | between Cj0887 & Cj0888 | Contains matches to CJIE3-like prophage, and hypothetical proteins; adjacent tRNA |
| 1336-Region 1B* | >24 | between Cj0887 & Cj0888 | Contains matches to CJIE3 ORFs; includes region with ORFs matching *Helicobacter* sp. proteins (mostly low identity matches); adjacent tRNA |
| 1336-Region 2 | 41 | between Cj0936 & Cj0937 | CJIE-2-like prophage; adjacent tRNA |
| 1336-Region 3 | 1.8 | between Cj0983 & Cj0984 | *Arcobacter butzleri* hypothetical membrane protein; possible sulphatase |
| 1336-Region 4 | 17 | between Cj1135 & Cj1149 | LOS locus |
| 1336-Region 5 | 27 | between Cj1305 & Cj1341 | Glycosylation locus, flagellar genes, putative motility accessory factors; includes ORFs matching non-*Campylobacter* proteins |
| 1336-Region 6 | 3.2 | between Cj1359 & Cj1361 | Restriction-modification locus |
| 1336 Region 7 | 26 | between CJ1414 & Cj1443 | Capsule-related proteins; contains ORFs with low identity matches to both *Campylobacter* and non-*Campylobacter* proteins |
| 1336-Region 8 | 12 | between Cj1548 & Cj1564 | Includes enzymes, RM and regulatory proteins; some ORFs matching non-*Campylobacter* proteins |
| 1336-PR1 | 13 | between Cj0288 & Cj0300 | includes novel CdtABC predicted proteins sharing 62-80% identity with *C. lari* proteins |
| 414-Region 1 | 69 | between Cj0653 and Cj0659 | CMLP1-like element; ORFs matching *C. coli*, including putative type VI secretion proteins; some ORFs matching non-*Campylobacter* proteins |
| 414-Region 2 | 11.8 | between Cj1544 & Cj1565 | includes putative enzymes, regulatory and transport and proteins; some ORFs matching non-*Campylobacter* proteins |
| 414-Region 3 | 7.4 | between Cj0508 & Cj0509 | includes some phage-related proteins, putative transcriptional regulator and putative sugar transferase |
|  |  |  |  |

*Relative positions of 1336 regions 1a and 1b are unconfirmed
