# Supplementary material for: Genomic variations define divergence of water/wildlife-associated Campylobacter jejuni niche specialists from common clonal complexes
Source: Environ Microbiol. 2011 Mar 21;13(6):1549–60. doi: 10.1111/j.1462-2920.2011.02461.x (PMC3569610; doi:10.1111/j.1462-2920.2011.02461.x)
Supplement: Supplementary file 10 [file emi0013-1549-sd10.doc]

**Table S6. Details of oligonucleotide primers for PCR assays**

| **PCR primers** | **Sequences (5’ to 3’)** | **Amplicon size (bp)** | **A. T. (^o^C)*** | **Comments on targeted region** |
| --- | --- | --- | --- | --- |
|  |  |  |  |  |
| **PCR assays for novel regions of the genome of strain 1336** | | | | |
| Con87orf10F  Con87orf10R | GAAGAGCGCATTACACAA  CTTGGCAGGGCATATGTA | 512 | 55 | 1336 region 1B; ORF (C1336_00024_7) shares 62% identity with *Helicobacter hepaticus* hypothetical protein (1a in Table 2) |
| Con87orf2F  Con87orf2R | GCACAGGGCAATAAAAAG  ACACCAATGCCCATACTC | 191 | 50 | 1336 region 1B; predicted ORF (C1336_00024_1) with no database matches (1b in Table 2) |
| Con15orf6F  Con15orf6R | GAATCCAATCCACCTCTC  GTTCATTATCACGCTTTTGC | 542 | 55 | 1336 region 1B ; ORF(C1336_00021-3) shares 31% identity with part of a *Helicobacter hepaticus* hypothetical protein (1c in Table 2) |
| Con45orf29F  Con45orf29R | GAAAGCGTGATCAAGTTG  CTTCCTATCGTTTAGCCTTA | 309 | 50 | 1336 region 1A; ORF (C1336_00080_5) shares 54% identity with 30S ribosomal protein S15 of *Helicobacter pullorum* (1d in Table 2) |
| Con87orf83F  Con87orf83R | TTACGGTGGAGACTATGA  ACCCTCTTCTAACAGTTC | 334 | 55 | 1336 region 2; ORF (C1336_00025_57) shares 36% identity with a *C. jejuni* subsp. *doylei* phage Lp1 protein |
| Con87orf139F  Con87orf139R | GATGAGCTTAACGCAGGA  GCCATAATACCGCCAAGC | 365 | 55 | 1336 region 2; ORF (C1336_00025_106) shares 50% identity with *C. jejuni* subsp. *doylei* hypothetical protein |
| Con32orf43F  Con32orf43R | TTTACGCATGCCTCAAGC  CGCGAGTCAAAACAGCAC | 227 | 55 | 1336 region 3; ORF (C1336_00025_153) shares 38% identity with an *Arcobacter butzleri* hypothetical membrane protein |
| Con64orf75F  Con64orf75R | TGCGATGCTTTGGGGTCA  GATACACGCCTACCCAAA | 676 | 55 | 1336 region 4; ORF (1336_00025_313) shares 72% identity with a transferase from a deep sea bacterium (4a in Table 2) |
| Con64orf90F  Con64orf90R | ATTGGAGTGATGTAGGGA  CCCCAAGGACGATGAGTA | 298 | 50 | 1336 region 4; ORF (C1336_00025_324) shares 56% identity with a predicted isomerise, ManC, of *C.* *curvus* (4b in Table 2) |
| Con34orf15F  Con34orf15R | GCTCGGTTATGTTATGGTA  TTCCTCCCCAAGTTTTCA | 266 | 55 | 1336 region 5; ORF (C1336_00027_13) shares 54% identity with a cytidyltransferase-related domain protein of *Desulfatibacillum alkenivorans* (5a in Table 2) |
| Con34orf20F  Con34orf20R | GTGCCCTCAGGTTTCTTG  ACTTGCATGAGCCCTAAA | 259 | 50 | 1336 region 5; ORF (C1336_00027_18) shares 24% identity with part of aminotransferase family protein of strain RM1221 (CJE0612) (5b in Table 2) |
| Con34orf25F  Con34orf25R | TGAAGGACATAGCGTAGC  CCCCTTCAGTACAGTTGT | 311 | 55 | 1336 region 5; ORF (C1336_00027_21) is a putative motility accessory factor (5c in Table 2) |
| Con8orf28F  Con8orf28R | GCTGTGCTATGTCTTACG  CTCTACGCACTAAGGCTA | 300 | 55 | 1336 region 6; ORF (C1336_00029_20) is a restriction-modification protein |
| Con10orf21F  Con10orf21R | CATTTGCCTTGGGATGAT  TAACCACCTCATCGCTTC | 592 | 55 | 1336 region 7; ORF (C1336_00031_10) shares 26% identity with part of a predicted hydrolase of *Helicobacter pylori* (7a in Table 2) |
| Con10orf4F  Con10orf4R | CTAGAATAGACTCGGCTA  AGCATCCCAAATGTCACA | 149 | 50 | 1336 region 7; ORF (C1336_00031_1) shares 32% identity with a predicted hydrolase of *Helicobacter pylori* (7b in Table 2) |
| Con29orf104F  Con29orf104R | AAGGCGCTAGAGATATTG  GCCTCCCATGAAATTCCA | 736 | 55 | 1336 region 8; ORF (C1336_00032_93) shares 35% identity with hypothetical protein of *Rhodococcus* sp. (8a in Table 2) |
| Con29orf117F  Con29orf117R | GGACTTGGTTGTATGGGA  CTCATACCCCAAGCTTTA | 410 | 50 | 1336 region 8; ORF (C1336_00032_104) shares 67% identity with a putative aldo/keto reductase of *Helicobacter pullorum* (8b in Table 2) |
| 1336-417F  1336-417R | CTTTTGGCGATGCAAATGTA  CATCATGTATCGGCGTTTGA | 231 | 55 | 1336 PR1; alternative *cdtA*-like gene (C1336_00006_29) |
|  |  |  |  |  |
| **PCR assays for novel regions of the genome of strain 414** | | | | |
| 414-531F  414-531R | CGTGGGCTTTACCAAGAAAA  CCTTTGCCTCCAAGTCCTAA | 221 | 62 | 414 region 1; ORF (C414_00008_15) shares 93% identity with a putative baseplate assembly protein V of *C. coli* |
| 414-543F  414-543R | CAATGGCGGACAATTTTTCT  ATGCTCCGGCTAAGCTATCA | 242 | 60 | 414 region 1; ORF (C414_00008_27) shares 94% identity with a phage uncharacterised protein of *C. jejuni* CF93-6 |
| 414-1856F  414-1856R | GTTTTGGCGGTTTTGCTTTA  CATTGCCGCTAAGTTTTTCC | 231 | 57 | 414 region 2; ORF (C414_00002_1) shares 72% identity with a hypothetical protein of *Helicobacter hepaticus* |
| 414-741F  414-741R | TGGCTGGGAGAAGAGAGGTA  TCATTACCACCACCAGCAGA | 192 | 60 | 414 region 3; ORF (C414_00008_205) shares 68% identity with a putative transcriptional regulator of *C. jejuni* RM1221 |
| 414-1602F  414-1602R | GAAACTCCTAGCGCTAAAG  GTCCATAACGCACRAAACG | 368 | 62 | within divergent 414 flagellin glycosylation locus; equivalent to Cj1324 of strain NCTC11168 but primers would not bind to the NCTC11168 gene |
|  |  |  |  |  |
| **PCR assays for deletions with respect to the genome of strain NCTC11168 (variable amplicon sizes)** | | | | |
| Cj1166F  Cj1168R | CCTCRGCTATTCTTCCTA  TGGGTGGCTATACTTGTA | - | 60 | L-lactate dehydrogenase |
| Cj0817F  Cj0820R | CCTGARTTTTGGGGTATAGC  GGCTATGGGTATGATGATG | - | 62 | Putative lipoprotein |
| Cj0551F  Cj0555R | GGATGCTTGATGGTATGA  GAGTTCCATTTACGGTTGC | - | 60 | Includes two putative membrane proteins |
| Cj0965F  Cj0976R | CCGCATCAGTATCTTCAT  CCAAGACAGAAAATCACATC | - | 60 | Putative integral membrane and periplasmic proteins |
| Cj0175F  Cj0182R2 | AGGAGCTGTAGCACTTCT  TAAGCCCATAAAGCCCAT | - | 62 | Iron transport proteins, including TonB protein and receptor |
| lct Pup  cdt dn | CTATAGGCCACATACCATTTG  ACTGYGCTCTTGACCAATC | - | 62 | *cdtABC* genes |
| 414-1601dn  414-1602up | CCTAGTAGCGTTAGAATA  CAGGTCTAGTATTTGGCAT | - | 55 | Flagellin glycosylation locus, primers are designed to strain NCTC11168 ORFs Cj1320 and Cj1324 |
|  |  |  |  |  |

* A.T.; annealing temperature
